# Household SARS-CoV-2 transmission during Omicron wave in Chiang Mai, Thailand: a prospective observational study

**DOI:** 10.1016/j.lansea.2025.100711

**Published:** 2026-01-05

**Authors:** Woottichai Khamduang, Pitaya Suebtam, Intira Jeannie Collins, Patumrat Sripan, Kittipan Chalom, Sayamon Hongjaisee, Nang Kham-Kjing, Nantawan Wangsaeng, Premmarin Inmonthian, Aphirak Pinasu, Napatsakorn Kohklang, Mathis Arnal, Moira Spyer, Ilse Steffens-Westerhof, Apinun Aramrattana, Marc Lallemant, Chaisiri Angkurawaranon, Patricia Bruijning-Verhagen, Nicole Ngo-Giang-Huong

**Affiliations:** a Department of Medical Technology, Faculty of Associated Medical Sciences, Chiang Mai University, Chiang Mai, Thailand; b LUCENT International Collaboration, Faculty of Associated Medical Sciences, Chiang Mai University, Chiang Mai, Thailand; c MIVEGEC - LMI PRESTO, French National Research Institute for Sustainable Development (IRD), Chiang Mai, Thailand; d MRC Clinical Trials Unit at UCL, University College London, United Kingdom; e Research Institute for Health Sciences, Chiang Mai University, Chiang Mai, Thailand; f Chiang Mai Provincial Health Office, Chaing Mai, Thailand; g AMS-PHPT Research Collaboration, Faculty of Associated Medical Sciences, Chiang Mai University, Chiang Mai, Thailand; h UCL Great Ormond Street Institute of Child Health, London, United Kingdom; i Department of Epidemiology, Julius Center for Health Sciences and Primary Care, University Medical Center Utrecht, Utrecht, the Netherlands; j Department of Family Medicine, Faculty of Medicine, Chiang Mai University, Chiang Mai, Thailand

**Keywords:** SARS-CoV-2, Household transmission, Omicron variant, Anti-NCP IgG, Whole-genome sequencing, Thailand, Children

## Abstract

**Background:**

SARS-CoV-2 transmission studies involving children in Thailand have been relatively limited to the early waves with the alpha and delta variants. Our study aims to address these gaps by examining household transmission in Chiang Mai, northern Thailand, during the Omicron wave in a post vaccination period.

**Methods:**

This prospective observational study enrolled households comprising a confirmed COVID-19 index patient with at least one uninfected contact and a child (<18 years of age who maybe an index or contact). Participant data, nasopharyngeal swabs, and blood samples were collected at entry and final visit. Participants recorded daily symptoms for 21 days and self-administered SARS-CoV-2 antigen tests every other day for 14 days. Incident infections were confirmed by RT-PCR. Secondary attack rates (SARs) were calculated and associated factors were analyzed using multivariable generalized estimating equations models. Phylogenetic analysis was used to confirm intra-household transmission.

**Findings:**

From July 2022 to May 2024, 93 households (93 index cases, 197 contacts) were enrolled; 52% of index cases and 49% of contacts were <18 years. Among contacts, despite 89% (175/197) having received the SARS-CoV-2 vaccine (of whom 75% > 6 months prior), 44 became infected, yielding a household SAR of 33% (95% CI: 24–44). In phylogenetically-confirmed transmission, SAR was 25% (95% CI: 17–35). Index low viral load (aRR: 0.82, 95% CI: 0.74–0.92) and contacts baseline anti-NCP IgG positivity (aRR: 0.42, 95% CI: 0.22–0.83) were significantly associated with lower household transmission.

**Interpretation:**

Despite widespread vaccination, household transmission of SARS-CoV-2 remained common. Prior immunity in contacts and lower viral load in index cases reduced risk. These findings underscore the central role of households in ongoing spread and highlight the value of booster vaccination and genomic surveillance to clarify transmission pathways and inform prevention policies.

**Funding:**

The study was funded by the 10.13039/100020655European Health and Digital Executive Agency (HADEA), 10.13039/501100000780European Commission, and by the 10.13039/100012947Institut de Recherche pour le Développement (IRD), France.

**Research in context**

**Evidence before this study**

We searched PubMed using the terms (“SARS-CoV-2” OR “COVID-19”) AND (“household transmission”) for articles published between January 2020 and December 2021. This time frame corresponds to when our study was initiated. This search identified 139 articles, of which few were prospective household studies conducted in Southeast Asia. During the subsequent Omicron wave, additional publications reported high secondary attack rates (SARs) across various settings. However, data on children-related transmission in post-vaccination settings remained limited, especially in Southeast Asia. Moreover, few studies have used genomic evidence to distinguish between intra-household and community-acquired transmission. Our study addresses these gaps by examining household transmission involving children in Chiang Mai, northern Thailand, during the Omicron wave.

**Added value of this study**

This prospective cohort study combines longitudinal clinical, serological, virological, and genomic data to investigate SARS-CoV-2 transmission within households during the Omicron wave. Despite high vaccine coverage, the study reveals that transmission occurred in one-third of households. Children and adults were equally likely to transmit the virus within the household. Whole genome sequencing shows that most infections were likely to be intra-household transmission. Notably, prior immunity reduced transmission risk to family members by more than half and a lower viral load in index cases was associated with reduced transmission risk to household members.

**Implications of all the available evidence**

This study highlights the critical need to prevent household infection, particularly in SEA where large family sizes and limited living spaces increase the risk. Our findings emphasize the continued importance of booster vaccination and early case isolation, even in the post-pandemic era when populations are highly immunized. Incorporating routine genomic surveillance into outbreak investigations is essential to refine contact tracing, clarify infection sources, and optimize control measures in COVID-19 management.

## Introduction

Thailand was the first country outside China to report a confirmed case of SARS-CoV-2 in January 2020,^1^ and has since experienced multiple waves of transmission.^2^^,^^3^ Thailand began the roll-out of SARS CoV-2 vaccines in early 2021, initially using inactivated virus vaccines (CoronaVac by Sinovac Biotech Ltd. and BBIBP-CorV by Sinopharm), and a viral vector vaccine (ChAdOx1 nCoV-19 by AstraZeneca). Thailand also adopted heterologous prime-boost regimens (mixed vaccine schedules) as part of its immunization strategy.^4^ From August 2021, mRNA-based vaccines were introduced for adults and adolescents and extended to younger children from February 2022.^5^^,^^6^ A modeling study based on Ministry of Public Health (MoPH) data estimated that vaccination prevented 84,518 deaths among individuals over 80 years old,^6^ highlighting the age-dependent protective effects of COVID-19 vaccines.

Chiang Mai Province is in the northern part of Thailand and has an estimated population of approximately 1.8 million people. The city is a major tourist hub and experienced significant economic disruption during the COVID-19 pandemic, particularly in its tourism-dependent sectors. In January 2021, the Chiang Mai University Mass Vaccination Hub (CMU-MVH) opened as part of Thailand's mass immunization campaign.^7^ Despite the widespread adoption of SARS-CoV-2 infection control measures in Thailand, transmissibility of new SARS-CoV-2 variants among Thai households remains high.^8^ Households remain indeed the primary setting for transmission and are considered significant drivers of the pandemic.^9^^,^^10^ Household transmission studies are essential for understanding secondary attack rates (SARs), transmission dynamics, and associated factors under real-world conditions. However, relatively few studies have assessed household transmission dynamics after the widespread rollout of SARS CoV-2 vaccines, particularly the impact of vaccines on household transmission.^11^^,^^12^

Furthermore, few studies have incorporated whole genome sequencing (WGS) to determine whether infections among household contacts are due to intra-household transmission or community-acquired infection. This distinction is critical for designing isolation measures and informing public health policies.^13^^,^^14^ Additionally, studies involving children have been relatively limited and were mostly conducted during the early waves with the alpha and delta variants.^15^^,^^16^ Our study addresses these gaps by examining household transmission in Chiang Mai, northern Thailand, during the Omicron wave. We aim to estimate the rate of SARS-CoV-2 transmission within households and identify associated risk factors. By integrating clinical, virological, immunological, and genomic data, we provide a comprehensive understanding of transmission dynamics during a period of high vaccine coverage.

## Methods

### Study design and population

Between July 2022 and May 2024, this prospective observational study enrolled households consisting of at least one uninfected contact and one child (<18 years of age) who could be either the index case or a contact at risk of infection. The study design, based on the European RECOVER household transmission study,^15^ was adapted to the Thai setting. The study included either urban or suburban households.

### Study population recruitment

Index cases diagnosed with SARS-CoV-2 were identified through the Chiangmai Provincial Public Health Office or primary healthcare hospitals. They were then referred to the study team for assessment of eligibility and interest in participation. Households were eligible for inclusion if they met the following criteria: (i) an index case with a positive SARS-CoV-2 RT-PCR or antigen result within the previous 48 h, (ii) residence in a household with ≥2 members, of whom ≥1 tested negative for SARS CoV-2 antigen at enrollment, and (iii) the household including ≥1 child (<18 years of age) participating in the study as the index case or a household contact. Once the index case agreed, the study team visited the household and conducted the consent process.

### Study procedures

#### At enrollment

A representative of each household completed a paper-based questionnaire about the household composition and living conditions. Each study participant completed a questionnaire about their sociodemographic characteristics, clinical history, and SARS CoV-2 vaccination history. Nasopharyngeal/throat swabs were collected from all household members who consented while peripheral blood samples were collected from contacts only. Nasopharyngeal/throat swabs were subsequently subjected to SARS-CoV-2 RT-PCR testing to confirm the infection status.

#### During follow-up

Household contacts were monitored for 21 days (Table S1). During this time, they self-administered SARS-CoV-2 antigen test using a rapid diagnostic test, RDT (Flowflex SARS-CoV-2 Antigen Rapid Test, ACON Biotech, Hangzhou, China) every other day for 14 days. They also reported daily clinical symptoms via mobile phone for 21 days and provided peripheral blood samples at the end of the follow-up visit. If a household contact had a positive antigen test (with or without symptoms), the study mobile team conducted a home visit and collected nasopharyngeal/throat swabs and blood samples on the day of positive results. Follow-up for that household was then extended for an additional 14 days. Blood samples were transported and processed on the same day. Nasopharyngeal/throat swabs and plasma samples were kept at −70 °C until use. At the end of the study, a household representative completed an end-of-study questionnaire.

### Laboratory analysis

#### Virological evaluations

Viral RNA was extracted from nasopharyngeal/throat swabs using the Zybio Nucleic Acid Extraction Kit B200-32T (Zybio Inc., Chongqing, China) and tested for the presence of SARS-CoV-2 *N* and *S* genes using Tellgen SARS-CoV-2 Nucleic acid detection kit (Tellgen Corp., Shanghai, China). Samples with a cycle threshold value ≤ 40 were defined as “detectable/positive”.

#### Immunological evaluations

All K_2_EDTA plasma samples collected from household contacts at baseline, at time of positive SARS-CoV-2 antigen, and at end of study were tested for qualitative IgG and IgM anti-SARS-CoV-2 nucleocapsid protein (NCP) using EUROIMMUN anti-SARS-CoV-2 NCP ELISA (EUROIMMUN, Lübeck, Germany). Results at each time point were reported as the ratio of the sample signal to the cut-off signal (S/CO ratio). If the S/CO ratio was <1.1 the sample was considered negative; if it was ≥1.1 the sample was considered positive for IgG or IgM. Analysis of the IgM S/CO ratio over the study period was conducted to identify an IgM seroconversion between the enrollment and end-of-study samples. This may reflect an infection that was not detected by the SARS-CoV-2 RDT or RT-PCR tests. The results of IgG S/CO ratio at enrollment were used for the analysis of factors associated with SARS-CoV-2 transmission.

#### Whole genome sequencing of SARS-CoV-2

Samples from all index cases and incident infections with a RT-PCR cycle threshold (Ct) value less than 30 underwent whole genome sequencing using Oxford Nanopore Technologies and the ARTIC SARS-CoV-2 sequencing protocol v4 (ARTIC v5.3.2, NCOV-2019 Panel: 10016495)^17^ (https://artic.network/). PCR products were indexed using the Native Barcoding Kit 24 V14 (SQK-NBD114.24). Sequencing was performed using a MinION MK1C with R10.4.1 flow cells (FLO-MIN114). Raw sequence reads (FASTQ files) were filtered and quality checked using fastp designed for short reads (300 and maximum limit acceptable of 600)^18^ and a Phred quality score of at least 10. FASTQ sequences were aligned using the open source basecaller for Oxford Nanopore reads (https://github.com/nanoporetech/dorado). The resulting BAM file was then submitted to SAMtools to sort and pileup the sequences and iVar was used to generate the consensus genome (FASTA sequence).^19^ Pangolin was used to assign SARS-CoV-2 lineages.^20^ All the processes were performed using Linux operating systems with Conda environment.

#### Phylogenetic analysis

After quality checking and duplication removal, remaining SARS CoV-2 whole genome sequences underwent phylogenetic analysis. An additional 1850 sequences downloaded from GISAID representing SARS-CoV-2 genomes from northern Thailand during the study period were used (https://gisaid.org/hcov19-variants). All sequences were aligned using MAFFT^21^ using the Wuhan Hu-1 reference genome (NC_045512.2). Then, a maximum likelihood phylogenetic tree was constructed using IQTREE v2.0^22^ with 1000 bootstrap replicates. Trees were then time-calibrated using TreeTime.^23^

To infer likely transmission events, we performed tree-based clustering using a genetic distance threshold corresponds to approximately 3 SNPs. Clustering was performed across all bootstrap trees to calculate a cluster probability for each sequence pair. Likely intra-household transmission was defined when viral genomes from index and secondary cases within the same household clustered together in more than 90% of bootstrap trees and community-acquired transmission was identified when sequences from secondary cases clustered separately or with unrelated community sequences. Phylogenetic trees and clustering results were visualized using the ggtree^24^ package in R.4.5.0.^25^ A detailed description of the phylogenetic analysis is provided in Table S2.

### Definition of SARS-CoV-2 secondary infection within the household

In the main analysis, a secondary infection was defined as a household contact who tested negative by both RT-PCR and RDT at study entry but subsequently tested positive to both tests during follow-up. In the sensitivity analyses, a secondary infection was defined as (i) a positive RT-PCR and RDT or an anti-nucleocapsid (anti-NCP) IgM seroconversion at the end of the study; or (ii) a positive RT-PCR and RDT with confirmation of intra-household transmission based on the phylogenetic analysis of SARS-CoV-2 whole genome sequences from infected household contacts and the index cases.

### Statistical analysis

**Fig. 1:**
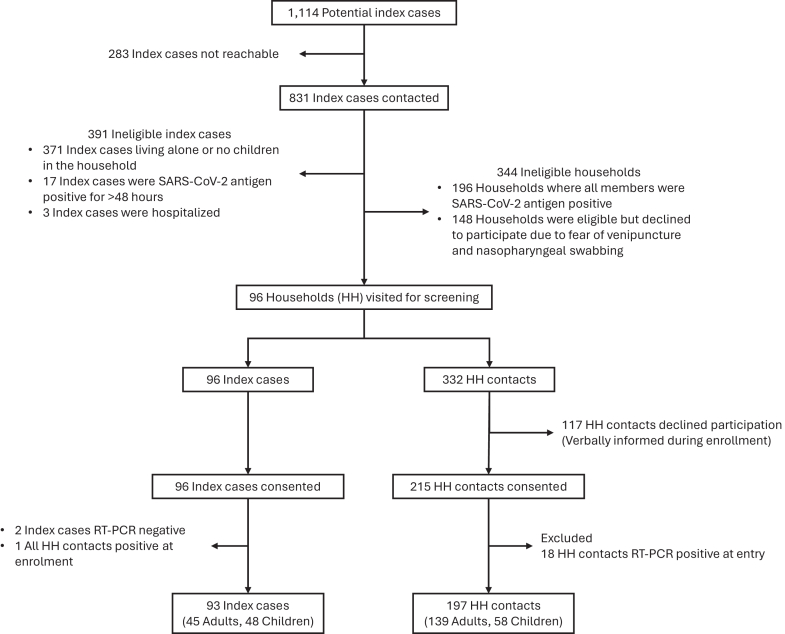
**Flowchart of household and participant enrollment**.

Descriptive statistics were used to summarize the baseline characteristics of households, index cases, and household contacts, stratified by the age of the index case (<18 and ≥ 18 years). Secondary attack rates (SARs) were calculated at the household and individual levels. The household-level SAR was defined as the proportion of households with ≥1 secondary infection among participating household contacts. The person-level SAR was defined as the proportion of secondary infections among all at risk household contact. Based on ethical considerations, household members were free to decide whether to participate in the study or to decline. Therefore, some households had all members consent to participate, while others had only partial participation depending on their willingness. The reasons for declining to participate were described (Fig. 1). The characteristics of households, index cases, and household members in the two groups were described in Tables S3–S5. Additionally, the factors associated with household SARS CoV-2 infection and the secondary attack rates were estimated for households with complete participation versus those with partial participation (Tables S6 and S7). Estimates were presented with 95% confidence intervals and stratified by the age of index cases. SAR results were plotted using the ggplot2^26^ package in R.

To identify factors associated with secondary SARS-CoV-2 infections, we used generalized estimating equations (GEE) to account for household clustering, using STATA 17.^27^ Included factors were household characteristics, index case characteristics (*e.g.* age, vaccination status, SARS CoV-2 RT-PCR Ct for *N* gene), at risk household contact characteristics (*e.g.* age, sex, vaccination status, comorbidities, SARS CoV-2 IgG S/CO ratio) at study entry. A log link function with an exchangeable correlation structure was applied in all GEE models. Variables with a p-value <0.20 in univariable analyses were included in the multivariable GEE models to identify independent risk factors of secondary infection and transmission. Age of the index case and household size were included in the final model as a priori confounders, based on previous studies,^15^^,^^28^ to account for their potential influence. Both main analyses and sensitivity analyses were conducted to assess the robustness of the associations. The results of multivariable models are presented as adjusted risk ratios (aRR) with 95% confidence intervals (95% CI) for the most relevant variables. Forest plots were generated using the ggplot2^26^ package to visualize the effect estimates. Statistical significance was defined as a p-value <0.05.

### Ethics statement

This study was approved on June 6th, 2022, by the Ethic Committee of the Faculty of Associated Medical Sciences, Chiang Mai University (AMSEC-65FB-004). Written informed consent was obtained from adults and from parents/legal guardians of enrolled children; assent was sought from children, where appropriate. All members of the household were invited to participate and only those who consented were included. Households were included if they met the above inclusion criteria, even if not all members agreed to participate.

### Role of the funding source

The funders had no role in study design, data collection, data analysis, interpretation, or writing of the report.

## Results

### Study population

The flow diagram of study population selection is shown in Fig. 1. Between July 2022 and May 2024, a total of 1114 potential index cases were identified for screening, of whom 831 were contacted. Among those contacted, only 96 households met the eligibility criteria (break up for non-eligibility provided in Fig. 1), 96 index cases and 332 household contacts were enrolled. Recruitment of index cases and the first household visit were typically conducted within 1–2 days after notification. After exclusion based on consent and positive status, final analysis included 93 households, comprising 93 index cases (45 adults and 48 children) and 197 at-risk household contacts (139 adults and 58 children).

### Baseline characteristics

Household, index case, and household contact characteristics are summarized in Table 1, Table 2, Table 3. The median household size was 4 persons (IQR: 3–5). Most households had two or more bedrooms (96%) (Table 1). The median age of the pediatric index cases was 11 (IQR: 5–13) and adult was 43 (IQR: 35–50), 69% of pediatric and 100% of adult index cases had received at least one dose of a SARS-CoV-2 vaccine, 48% of pediatric and 9% of adults received vaccine less than 6 months prior to enrollment (Table 2).

**Table 1:** Characteristics of households, overall and by index age group.

Household characteristics	Total (n = 93)	Index case age <18 years (n = 48)	Index case age ≥18 years (n = 45)
Household size (including index cases), median (IQR)	4 (3–5)	4 (3–5)	4 (4–5)
Range (min–max)	2–8	2–7	3–8
≤4 persons, n (%)	52 (56)	27 (56)	25 (56)
>4 persons, n (%)	41 (44)	21 (44)	20 (44)
Number of bedrooms, median (IQR)	3 (2–3)	3 (2–3)	3 (2–3)
1 room, n (%)	4 (4)	4 (8)	0 (0)
2 rooms, n (%)	35 (38)	17 (35)	18 (40)
3 rooms, n (%)	38 (41)	17 (35)	21 (47)
4 rooms, n (%)	14 (15)	9 (19)	5 (11)
5 rooms, n (%)	1 (1)	1 (2)	0 (0)
7 rooms, n (%)	1 (1)	0 (0)	1 (2)
Number of bathrooms, median (IQR)	2 (1–2)	2 (1–2)	2 (1–3)
1 room, n (%)	35 (38)	18 (38)	17 (37)
2 rooms, n (%)	38 (41)	22 (46)	16 (37)
3 rooms, n (%)	15 (16)	5 (10)	10 (22)
4 rooms, n (%)	4 (4)	2 (4)	2 (4)
5 rooms, n (%)	1 (1)	1 (2)	0 (0)
Household members wearing masks indoors, n (%)	77 (83)	40 (83)	37 (82)
Air condition use	20 (22)	14 (29)	6 (13)

**Table 2:** Characteristics and laboratory results of index cases, overall and by index age group.

Index case characteristics	Total (n = 93)	Index case age <18 years (n = 48)	Index case age ≥18 years (n = 45)
Male	46 (50)	27 (56)	19 (42)
Age in years, median (IQR)	17 (11–43)	11 (5–13)	43 (35–50)
Children (<18 years), n (%)	48 (52)	48 (100)	0 (0)
Adult (18–59 years), n (%)	40 (43)	0 (0)	40 (89)
Elderly (>60 years), n (%)	5 (5)	0 (0)	5 (11)
Highest Education level in adults, n (%)	n = 45	n = 0	n = 45
Primary school or below	10 (22)	–	10 (22)
High school	20 (44)	–	20 (44)
Bachelor's degree or higher	15 (33)	–	15 (33)
BMI^a^, n (%)	n = 93	n = 48	n = 45
Normal	41 (44)	28 (58)	13 (29)
Underweight	3 (3)	2 (4)	1 (2)
Overweight	49 (53)	18 (38)	31 (69)
Have any symptoms, n (%)	89 (96)	45 (94)	44 (98)
SARS-CoV-2 vaccine doses received, n (%)	n = 93	n = 48	n = 45
None	15 (16)	15 (31)	0 (0)
1–2 doses	35 (38)	22 (46)	13 (29)
≥3 doses	43 (46)	11 (23)	32 (71)
SARS-CoV-2 vaccine type received, n (%)	n = 78	n = 33	n = 45
mRNA only	37 (47)	33 (100)	4 (9)
mRNA + other	12 (15)	0 (0)	12 (27)
Non mRNA	29 (37)	0 (0)	29 (64)
Most recent vaccine type received, n (%)	n = 78	n = 33	n = 45
Inactivated	1 (1)	0 (0)	1 (2)
Vector	9 (12)	0 (0)	9 (20)
mRNA	68 (87)	33 (100)	35 (78)
Median time since last vaccine dose, n (%)	n = 78	n = 33	n = 45
Less than 1 month	1 (1)	0 (0)	1 (2)
1–3 months	2 (23)	2 (6)	0 (0)
4–6 months	17 (22)	14 (42)	3 (7)
More than 6 months	58 (74)	17 (52)	41 (91)
Index cases wearing masks indoors, n (%)	85 (91)	43 (90)	42 (93)
Index cases using a separate bed from other household members, n (%)	78 (84)	39 (81)	39 (87)
**Laboratory results**			
Lineage of SARS CoV-2, n (%)			
Omicron (BA)	56 (60)	31 (65)	25 (56)
Omicron (XBB)	23 (25)	7 (15)	16 (36)
Unspecified	14 (15)	10 (21)	4 (9)
RT-PCR cycle thresholds			
N gene, median (IQR)	25.2 (22.2–28.3)	26.1 (23.0–29.0)	23.5 (21.7–27.0)
N gene Ct < 30, n (%)	77 (84)	38 (81)	39 (87)
S gene, median (IQR)	22.9 (21.0–26.4)	24.2 (21.9–27.6)	22.0 (20.4–24.7)
S gene Ct < 30, n (%)	84 (91)	41 (87)	43 (96)

**Table 3:** Characteristics and laboratory results of household contacts, total and by index age group.

Household contacts characteristics	Total (n = 197)	Index case age< 18 years (n = 96)	Index case age ≥18 years (n = 101)
Male	105 (53)	51 (53)	54 (54)
Age, median (IQR)	36 (15–52)	39 (30–56)	27 (12–45)
Children (<18 years), n (%)	58 (29)	13 (14)	45 (41)
Adult (18–59 years), n (%)	106 (54)	65 (68)	41 (41)
Elderly (>60 years), n (%)	33 (17)	18 (19)	15 (15)
Relation with index case, n (%)			
Child/Grandchild	12 (6)	1 (1)	11 (11)
Parent	64 (32)	48 (50)	16 (16)
Grandparent	21 (11)	21 (22)	0 (0)
Spouse	22 (11)	1 (1)	21 (21)
Other	78 (40)	25 (26)	53 (52)
Highest education level of adults, n (%)	n = 139	n = 83	n = 56
Primary school or below	43 (31)	24 (29)	19 (34)
High school	44 (32)	24 (29)	20 (36)
Bachelor's degree or higher	52 (37)	35 (42)	17 (30)
BMI^a^, n (%)			
Normal	95 (48)	42 (44)	53 (52)
Underweight	10 (5)	2 (2)	8 (8)
Overweight	92 (47)	52 (54)	40 (40)
Have Chronic diseases, n (%)			
Hypertension	32 (16)	20 (21)	12 (12)
Kidney disease	2 (1)	1 (1)	1 (1)
Diabetes	6 (3)	3 (3)	3 (3)
Smoking, n (%)	n = 139	n = 83	n = 56
Never	107 (77)	63 (76)	44 (79)
Ever smoking	13 (9)	9 (11)	4 (7)
Currently smoking	19 (14)	11 (13)	8 (14)
SARS-CoV-2 vaccine doses received, n (%)			
None	22 (11)	3 (3)	19 (19)
1–2 doses	58 (30)	26 (27)	32 (32)
≥3 doses	117 (60)	67 (70)	50 (50)
Vaccine type received, n (%)	n = 175	n = 93	n = 82
mRNA only	47 (27)	14 (15)	33 (40)
mRNA + other	39 (22)	26 (28)	13 (16)
Non mRNA	89 (51)	53 (57)	36 (44)
Latest vaccine type received, n (%)	n = 175	n = 93	n = 82
Inactivated	8 (5)	6 (6)	2 (2)
Vector	25 (14)	16 (17)	9 (11)
mRNA	142 (81)	71 (76)	71 (87)
Time from the latest vaccine dose, n (%)	n = 175	n = 93	n = 82
Less than 1 month	4 (2)	3 (3)	1 (1)
1–3 months	11 (6)	9 (10)	2 (2)
4–6 months	28 (16)	20 (22)	8 (10)
More than 6 months	132 (76)	61 (65)	71 (87)
Previously SARS CoV-2 positive, n (%)			
Yes	95 (52)	47 (49)	48 (48)
No	102 (48)	49 (51)	53 (52)
Contacts wearing masks indoors, n (%)	77 (83)	40 (83)	37 (82)
**Laboratory results**			
NCP IgG S/CO ratio at baseline (n = 176), median (IQR)	0.35 (0.08–1.03)	0.43 (0.07–1.33)	0.33 (0.09–0.76)
Positive (≥1.1), n (%)	43 (24)	29 (32)	14 (16)
Negative (<1.1), n (%)	133 (76)	61 (68)	72 (84)
NCP IgM S/CO ratio at baseline (n = 176), median (IQR)	0.15 (0.08–0.30)	0.14 (0.07–0.30)	0.15 (0.08–0.30)
Positive (≥1.1), n (%)	5 (3)	3 (3)	2 (2)
Negative (<1.1), n (%)	171 (97)	87 (97)	84 (98)

Children's BMI: Normal (z-score: < −2), underweight (z-score: −1.5 to 1.5), and overweight (z-score: >1.5).

a Adult's BMI: Normal (BMI: 18.5–22.9), underweight (BMI <18.5) and overweight (BMI: ≥23.0).

The contacts of pediatric index cases were primarily parents and grandparents (72%). The contacts had a high proportion of overweight individuals, with 25% of pediatric and 16% of adult index case contacts reporting chronic diseases. Among pediatric index case contacts 97% had received at least one SARS-CoV-2 vaccine with 35% receiving less than six months prior. Among contacts of adult index cases 82% received a SARS-CoV-2 vaccine with 13% receiving less than six months prior to enrollment (Table 3).

At enrollment, more than 80% of pediatric and adult index cases had *N* gene and *S* gene Ct < 30. Since both *N* and *S* genes Ct values reflect the viral replication and are highly correlated, only *N* gene Ct values were used for the analysis of factors associated with secondary infection (Table 2). 32% among pediatric index case contacts and 16% among adult index case contacts tested positive for prior immunity (anti-NCP IgG) (Table 3).

### Laboratory results

During follow up, 31 of the 93 households experienced secondary infections, resulting in 44 infected and 153 non-infected household members whose characteristics are summarized in Table S8. The median time between the index case's positive test or symptom onset and the confirmed infection of secondary cases was 4.5 days (IQR 3.0–5.0). Among 93 index cases and 44 household infections, 11 index cases and 4 SARS-CoV-2–positive household contacts had no sequence available due to low viral load (Ct Value ∼30). It leads us to a total of 122 individuals being sampled. After quality checking, we have 121 SARS-CoV-2 whole genome sequences of individuals (81 index and 40 contacts in 30 households). All these sequences were Omicron with 3 main Pango Lineages: BA.2 (38%), BA.5 (28%) and XBB (29%), the remaining being other Omicron lineages. In addition, 1850 sequences from GISAID have been added to these sequences to build the phylogenetic tree (Figure S1). The phylogenetic tree clustering identified 28 likely household transmissions in 23 households, and 12 community transmissions in 10 households, including three households with both types of transmissions.

### Household-level and individual-level secondary attack rates

**Fig. 2:**
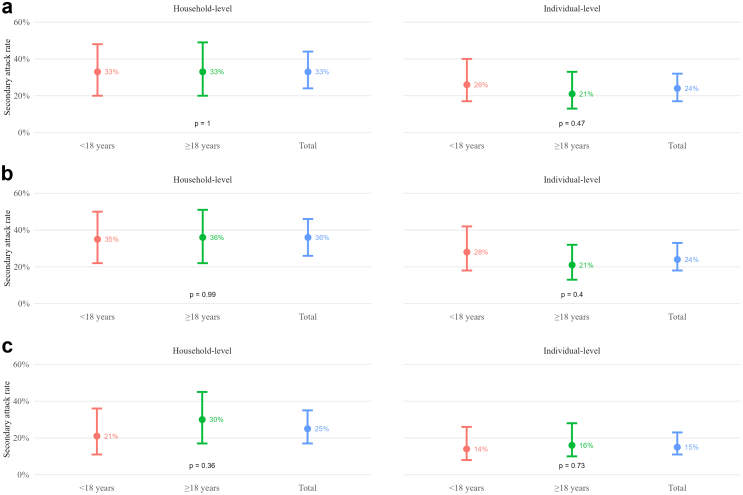
**Household and individual secondary attack rates (SARs) by index case age**. Panels a–c shows SARs according to the age of the index case: a) Main analysis based on RT−PCR positivity (n = 93 households, 197 individuals; household−level p = 1.000, individual−level p = 0.470). b) Sensitivity analysis based on RT−PCR or SARS−CoV−2 IgM positivity (n = 93 households, 197 individuals; household−level p = 0.990, individual−level p = 0.400). c) Sensitivity analysis based on RT−PCR and phylogenetic analysis (n = 91 households, 192 individuals; household−level p = 0.360, individual−level p = 0.730). Error bars represent 95% confidence intervals. SAR, secondary attack rate; RT−PCR, reverse transcription polymerase chain reaction.

The household-level SAR was 33% (95% CI: 24–44) and the individual-level SAR was 24% (95% CI: 17–32). A sensitivity analysis with secondary infections based on PCR or IgM seroconversion resulted in slightly increased household (36%; 95% CI: 26–46) and individual (24%; 95% CI: 18–33) -level SARs. A further sensitivity analysis restricted the secondary infections to those supported by PCR results and confirmed intra-household transmission using sequencing data. This resulted in household- and individual-level SARs of 25% (95% CI: 17–35), and 15% (95% CI: 11–23), respectively. In both the main and sensitivity analyses the household- and individual-level SAR did not significantly differ by age of the index case (Fig. 2).

### Factors associated with household SARS-CoV-2 infection

**Fig. 3:**
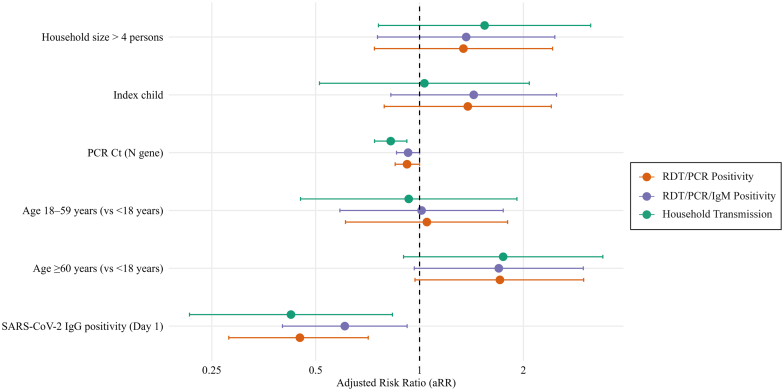
**Adjusted risk ratios (aRR) for SARS-CoV-2 transmission**. Forest plot showing aRRs for three outcomes: RDT/PCR positivity, RDT/PCR/IgM positivity, and confirmed household transmission. Variables include household size, index case N gene RT-PCR Ct value, household contact SARS-CoV-2 IgG positivity, and index case age.

The factors associated with household infection of SARS CoV-2 (main analysis), positive RDT/PCR or positive SARS CoV-2 IgM (sensitivity analysis i), and household transmission (sensitivity analysis ii) are shown in Tables S9–S11, respectively. The univariable analysis indicated that the index case's vaccination status, the *N*-gene RT-PCR Ct value, the age group of household contacts, with elderly contacts showing higher risk compared with children—and IgG positivity were each associated with transmission. The SARS-CoV-2 XBB lineage was not associated with a higher risk of infection compared to BA lineage. In the multivariable analysis (Fig. 3; Table S9), after adjusting for household size and the age groups of index cases and contacts, IgG positivity remained associated with a 55% lower risk of transmission (aRR: 0.45; 95% CI: 0.28–0.71; p = 0.001). The index case *N* gene RT-PCR Ct value was also associated with transmission risk (aRR: 0.92; 95% CI: 0.85–1.00; p = 0.045). Older household members tended to have a higher risk of infection than children (aRR: 1.71; 95% CI: 0.97–2.99; p = 0.062). A sensitivity analysis with secondary infections based on PCR or IgM seroconversion showed consistent results (Fig. 3; Table S10). The sensitivity analysis restricted to phylogenetically confirmed intra-household transmission showed an 18% reduction risk per one unit increase of *N* gene RT-PCR Ct value (aRR: 0.82; 95% CI: 0.74–0.92, p < 0.0001). Regarding household contact characteristics, IgG positivity was significantly associated with a 58% reduced risk of transmission (aRR: 0.42; 95% CI: 0.22–0.83; p = 0.013) (Fig. 3; Table S11).

## Discussion

This prospective household transmission study, conducted during the Omicron wave in Chiangmai, Thailand, offers important insights into SARS-CoV-2 transmission dynamics in the post-vaccine rollout era. At the beginning of the COVID-19 pandemic in Thailand, SARS-CoV-2 transmission was relatively well controlled. Individuals living in the same household as a COVID-19 patient were considered to have high-risk exposure. A serology study conducted among unvaccinated household contacts in Bangkok reported a seroprevalence of anti–SARS-CoV-2 S1 antibodies of 20.5%. Factors strongly associated with seropositivity included being related to the index case, staying in the same room, and engaging in shared leisure activities.^29^

Only a few household studies have been conducted in Thailand, most of which relied on retrospective analyses of data collected by the Ministry of Public Health during the early waves of the pandemic.^8^^,^^30^ In a retrospective case–control study from April 2020, which primarily used contact-tracing data from the Bangkok area, factors independently associated with a lower risk of infection included consistent mask use during contact, maintaining a distance of more than one meter from an infected person, having close contact for less than 15 min, and frequent handwashing. In that study, the household secondary attack rate was 16.5% (38/230).^30^ These findings highlight the importance of consistent mask wearing, hand hygiene, and physical distancing in preventing COVID-19 transmission. However, following the rollout of vaccination programs, strict isolation measures were relaxed, contributing to a lower perceived risk and decreased adoption of protective behaviors.

We found that SARS-CoV-2 secondary transmission remained substantial despite high vaccine rates and prior exposure in the community. The overall SAR was 33% (95% CI: 24–44) at the household level and 24% (95% CI: 17–32) at the individual level based on RT-PCR-confirmed cases. Sensitivity analyses incorporating anti-NCP IgM seroconversion increased SAR marginally, while WGS–confirmed intra-household transmission yielded a household-level SAR of 25% (95% CI: 17–35). These results are consistent with Omicron-era findings from Denmark^11^ but are lower than pre-vaccine SARs reported in Thai and Bhutanese studies conducted during earlier waves.^8^^,^^31^ A meta-analysis of household secondary attack rates (SARs) across variant waves, including 135 studies in 36 countries, reported the highest SAR during the Omicron period (42.7%; 95% CI, 35.4%–50.4%) and the lowest total vaccine effectiveness (35.8%; 95% CI, 13.0%–52.6%).^32^ Fully vaccinated contacts generally had lower SARs (18.8%) compared with unvaccinated contacts (36.5%; p < 0.0001); however, during the Omicron wave, SARs did not differ significantly between unvaccinated and booster-vaccinated contacts. This meta-analysis underscores the increased household transmissibility of newer variants and the reduced vaccine effectiveness against Omicron compared with earlier variants. Nonetheless, the duration of protection against emerging variants remains uncertain. The high overall secondary attack rate (SAR) is consistent with the known biological features of Omicron, including increased transmissibility and partial escape from vaccine-induced immunity,^32^ which allow breakthrough infections and maintain substantial onward transmission.

A key finding of our study is that the pre-existing immunity to SARS-CoV-2 reduces the risk of infection by 55% (aRR: 0.45; 95% CI: 0.30–0.69), indicating partial immunity derived from prior infection and/or vaccination. This finding is consistent with emerging evidence that hybrid immunity provides enhanced protection against Omicron infection.^33^ Viral load, approximated by *N* gene RT-PCR Ct value of index cases, was another key determinant of household transmission. We observed a significant inverse association between Ct value and risk of transmission in sensitivity models using phylogenetically-confirmed transmissions, with each unit increase in Ct value associated with a 18% reduction in transmission risk (aRR: 0.82; 95% CI: 0.74–0.92). This finding underscores the role of viral kinetics in modulating infectiousness.^34^ Contrary to some previous reports suggesting higher transmissibility among young children,^35^ our study found no statistically significant difference in SAR based on index case age, supporting inclusive surveillance and mitigation strategies regardless of age. In contrast, the age group of household contacts showed a trend toward differential susceptibility, with elderly contacts exhibiting a higher risk of acquiring infection compared with children.

A strength of our study is its comprehensive integration of clinical, virological, serological, and genomic data to robustly characterize transmission events. Phylogenetic analysis provided an essential layer of evidence for distinguishing intra-household transmission from community-acquired infections. By integrating genomic data with collection dates, we were able to characterize transmission pathways more confidently and reduce potential misclassification. These findings emphasize the importance of routine genomic sequencing in household studies and justify continued investment in genomic surveillance to improve our understanding of SARS-CoV-2 transmission dynamics. The intensive testing schedule and high participant retention (>95%) also strengthen the validity of our findings. However, several limitations should be acknowledged. About one third of household members did not consent to participate. We conducted a sub-analysis which included households with full participation (35 households) and partial participation of household members (58 households) (Tables S6 and S7) and found no difference in household level SARs (29% versus 36%, p = 0.45), while the overall SAR in the main analysis was 33% among 93 households. The protocol mentioned the criteria for inclusion, and it was possible to enroll households even though not all members consented. This highlights the challenge of achieving full household enrollment in field settings. Declining participation in fear of venipuncture should be considered when developing future studies. Of note, 18 household contacts were RT-PCR positive at entry. This may have influenced transmission patterns. Additionally, genomic sequencing could not be performed for all RT-PCR-positive cases, especially those with high Ct values, preventing the identification of the route of transmission. Furthermore, the use of anti-NCP IgG/IgM as a marker of past infection may be confounded by prior vaccination with whole-virion inactivated vaccines, which include the nucleocapsid protein and can elicit anti-NCP antibodies. However, the immune response to the nucleocapsid antigen is typically weaker and more transient compared to spike-based responses (*e.g.* anti-S, anti-RBD, or neutralizing antibodies), potentially leading to undetectable anti-NCP IgG in vaccinated individuals depending on the timing or type of prior vaccination. Thus, caution is needed when interpreting anti-NCP results as evidence of natural infection. Finally, this study was conducted in collaboration with the Chiang Mai Provincial Public Health Office and primary healthcare hospitals and relied on a mobile unit for sample collection. While this approach may limit the generalizability of our findings to other settings, it enabled access to participants who might not attend general hospitals and thus provided a sample more representative of the general population.

In conclusion, our findings demonstrate that despite a release of isolation and control measures, household transmission of Omicron SARS-CoV-2 remains a key concern even with high levels of vaccination. Prior immunity, as evidenced by anti-NCP IgG positivity, and lower viral load in index cases were associated with reduced transmission risk. These results highlight the role of households in ongoing transmission and reinforce the need for layered prevention strategies, including booster vaccination and isolation of high viral load individuals. Furthermore, genomic surveillance should be considered a routine component of outbreak investigations to accurately identify transmission pathways and guide prevention strategies and policy.

## Contributors

WK, IJC, IS-W, AA, ML, CA, PB, NN contributed on conceptualization. WK, IJC, PSr, IS-W, AA, MS, ML, CA, PB, NN developed the study methodology. WK, PSu, SH, NK-K, NW, PI, AP, NK, NN conducted the investigation. WK, IJC, KC, AA, ML, CA, PB, NN supervised the study. PSu, KC, SH, NK-K, NW, PI, AP, NK curated the data. WK, PSu, PSr, AP, NK, MA, and NN have accessed and verified the data. WK, PSu, PSr, MA and NN performed data analyses. WK, IJC, ML, MS, CA, PB, NN provided resources. WK, PSu, SH, PI, AP, NK, NN administrated the project. PSu, PSr, and MA prepared the visualizations. WK, PSu, IJC, PSr, MA, and NN drafted the manuscript. All authors contributed to interpretation of the results, revised the manuscript critically for important intellectual content, and approved the final version for submission. WK, PSr, MA, and NN were responsible for the decision to submit the manuscript.

## Data sharing statement

The 132 raw Nanopore sequencing reads and consensus sequences generated in this study have been deposited in the European Nucleotide Archive (ENA) under accession number PRJEB104853. The datasets used and/or analyzed during the current study are available from the corresponding author upon reasonable request.

## Statement on AI use

We used ChatGPT (GPT-5, OpenAI) to help with language editing and paraphrasing. All content was then reviewed and verified by the authors.

## Declaration of interests

The authors declare that they have no conflict of interests.

## Appendix A Supplementary data

Supplementary Materials

Abstract

## References

[1] Novel coronavirus – Thailand. https://www.who.int/emergencies/disease-outbreak-news/item/2020-DON234.

[2] Mahasirimongkol S., Uppapong B., Puangtubtim W. (2022). SARS-CoV-2 Seroprevalence in unvaccinated adults in Thailand in november 2021. Vaccines.

[3] Naemiratch B., Schneiders M.L., Poomchaichote T. (2022). “Like a wake-up call for humankind”: views, challenges, and coping strategies related to public health measures during the first COVID-19 lockdown in Thailand. PLOS Glob Public Health.

[4] Promlek T., Hansirisathit T., Kunno J., Thanunchai M. (2023). The effects of CoronaVac and ChAdOx1 nCoV-19 in reducing severe illness in Thailand: a retrospective cohort study. Trop Med Infect Dis.

[5] Kitro A., Sirikul W., Dilokkhamaruk E. (2022). COVID-19 vaccine hesitancy and influential factors among Thai parents and guardians to vaccinate their children. Vaccine X.

[6] Wilasang C., Suttirat P., Wannigama D.L., Amarasiri M., Chadsuthi S., Modchang C. (2024). Impact of COVID-19 vaccination in Thailand: averted deaths and severe infections across age groups. Trop Med Infect Dis.

[7] Kitro A., Tippong D., Sirikul W. (2025). Efficiency and simulation of Thailand's Chiang Mai university model for COVID-19 mass vaccination hub (CMU-MVH model). Am J Infect Control.

[8] Watanapokasin N., Siripongboonsitti T., Ungtrakul T. (2021). Transmissibility of SARS-CoV-2 variants as a secondary attack in Thai households: a retrospective study. IJID Reg.

[9] Leclerc Q.J., Fuller N.M., Knight L.E., Funk S., Knight G.M., CMMID COVID-19 Working Group (2020). What settings have been linked to SARS-CoV-2 transmission clusters?. Wellcome Open Res.

[10] Sun K., Wang W., Gao L. (2021). Transmission heterogeneities, kinetics, and controllability of SARS-CoV-2. Science.

[11] Lyngse F.P., Mortensen L.H., Denwood M.J. (2022). Household transmission of the SARS-CoV-2 omicron variant in Denmark. Nat Commun.

[12] Winkel A.M.A.M., Kozanli E., Haverkort M.E. (2025). Lower levels of household transmission of SARS-CoV-2 omicron variant of concern vs wild type: an interplay between transmissibility and immune status. J Infect Dis.

[13] Derqui N., Koycheva A., Zhou J. (2023). Risk factors and vectors for SARS-CoV-2 household transmission: a prospective, longitudinal cohort study. Lancet Microbe.

[14] Banga J., Brock-Fisher T., Petros B.A. (2024). Severe acute respiratory syndrome Coronavirus 2 household transmission during the Omicron era in Massachusetts: a prospective, case-ascertained study using genomic epidemiology. Open Forum Infect Dis.

[15] Verberk J.D.M., de Hoog M.L.A., Westerhof I. (2022). Transmission of SARS-CoV-2 within households: a remote prospective cohort study in European countries. Eur J Epidemiol.

[16] Bhatt M., Plint A.C., Tang K. (2022). Household transmission of SARS-CoV-2 from unvaccinated asymptomatic and symptomatic household members with confirmed SARS-CoV-2 infection: an antibody-surveillance study. CMAJ Open.

[17] Quick J., Lansdowne L. (2024). ARTIC SARS-CoV-2 sequencing protocol v4 (LSK114). https://www.protocols.io/view/artic-sars-cov-2-sequencing-protocol-v4-lsk114-dbw62phe.

[18] Chen S. (2023). Ultrafast one-pass FASTQ data preprocessing, quality control, and deduplication using fastp. Imeta.

[19] Danecek P., Bonfield J.K., Liddle J. (2021). Twelve years of SAMtools and BCFtools. Gigascience.

[20] O'Toole Á., Scher E., Underwood A. (2021). Assignment of epidemiological lineages in an emerging pandemic using the pangolin tool. Virus Evol.

[21] Katoh K., Standley D.M. (2013). MAFFT multiple sequence alignment software version 7: improvements in performance and usability. Mol Biol Evol.

[22] Minh B.Q., Schmidt H.A., Chernomor O. (2020). IQ-TREE 2: new models and efficient methods for phylogenetic inference in the genomic era. Mol Biol Evol.

[23] Sagulenko P., Puller V., Neher R.A. (2018). TreeTime: Maximum-likelihood phylodynamic analysis. Virus Evol.

[24] Xu S., Li L., Luo X. (2022). Ggtree: a serialized data object for visualization of a phylogenetic tree and annotation data. iMeta.

[25] R Core Team (2025). https://www.R-project.org/.

[26] Wickham H. (2009). https://link.springer.com/10.1007/978-0-387-98141-3.

[27] (2025). Stata statistical software.

[28] Metlay J.P., Haas J.S., Soltoff A.E., Armstrong K.A. (2021). Household transmission of SARS-CoV-2. JAMA Netw Open.

[29] Atsawawaranunt K., Thiangthangthum K., Sirikhetkon S. (2023). Seroprevalence of anti-SARS-CoV-2 antibodies and associated factors among household contacts of COVID-19 confirmed cases in Bangkok, Thailand. Heliyon.

[30] Doung-ngern P., Suphanchaimat R., Panjangampatthana A. (2020). Case-control study of use of personal protective measures and risk for SARS-CoV 2 infection, Thailand. Emerg Infect Dis.

[31] Jatsho J., Pelzom D., Dorji S., Pelzang T. (2022). Household transmission of SARS-CoV-2 in Bhutan. Biomed Res Int.

[32] Madewell Z.J., Yang Y., Longini I.M., Halloran M.E., Dean N.E. (2022). Household secondary attack rates of SARS-CoV-2 by variant and vaccination status: an updated systematic review and meta-analysis. medRxiv.

[33] da Silva M.F.B., Guaraldo L., Bastos L.S. (2025). Natural, vaccine-induced immunity and the probability of experiencing SARS-CoV-2 infection in a household cohort in Rio de Janeiro. Sci Rep.

[34] Jones J.E., Le Sage V., Lakdawala S.S. (2021). Viral and host heterogeneity and their effects on the viral life cycle. Nat Rev Microbiol.

[35] Reukers D.F.M., van Boven M., Meijer A. (2022). High infection secondary attack rates of severe acute respiratory syndrome Coronavirus 2 in Dutch households revealed by dense sampling. Clin Infect Dis.

