# Supplementary material for: Household SARS-CoV-2 transmission during Omicron wave in Chiang Mai, Thailand: a prospective observational study
Source: Lancet Reg Health Southeast Asia. 2026 Jan 5;44:100711. doi: 10.1016/j.lansea.2025.100711 (PMC12810560; doi:10.1016/j.lansea.2025.100711)
Supplement: Supplementary Materials [file mmc1.docx]

**Supplementary Tables**

**Table S1: study procedures**

| Day | 1 | 2 | 3 | 4 | 5 | 6 | 7 | 8 | 9 | 10 | 11 | 12 | 13 | 14 | 15 | 16 | 17 | 18 | 19 | 20 | 21 | 22 | 23 | 24 |
| --- | --- | --- | --- | --- | --- | --- | --- | --- | --- | --- | --- | --- | --- | --- | --- | --- | --- | --- | --- | --- | --- | --- | --- | --- |
| House visit | ✓ |  |  |  |  |  |  |  |  |  |  |  |  |  |  |  |  |  |  |  | ✓ |  |  |  |
| Consent | △  ● |  |  |  |  |  |  |  |  |  |  |  |  |  |  |  |  |  |  |  |  |  |  |  |
| Questionnaire | △  ● |  |  |  |  |  |  |  |  |  |  |  |  |  |  |  |  |  |  |  | ● |  |  |  |
| Nasopharyngeal/  throat swabs | △  ● |  |  |  |  |  |  |  |  |  |  |  |  |  |  |  |  |  |  |  |  |  |  |  |
| Peripheral blood | ● |  |  |  |  |  |  |  |  |  |  |  |  |  |  |  |  |  |  |  | ● |  |  |  |
| Daily symptoms | ● | ● | ● | ● | ● | ● | ● | ● | ● | ● | ● | ● | ● | ● | ● | ● | ● | ● | ● | ● | ● |  |  |  |
| Antigen test | △  ● |  | ● |  | ● |  | ● |  | ● |  | ● |  | ● |  |  |  |  |  |  |  | ● |  |  |  |
| If a contact tested antigen “positive” during follow up, for example at day 11, all HH contacts will continue follow up as below: | | | | | | | | | | | | | | | | | | | | | | | | |
| House visit |  |  |  |  |  |  |  |  |  |  | ✓ |  |  |  |  |  |  |  |  |  |  |  |  | ✓ |
| Questionnaire |  |  |  |  |  |  |  |  |  |  |  |  |  |  |  |  |  |  |  |  |  |  |  | ● |
| Nasopharyngeal/  throat swabs |  |  |  |  |  |  |  |  |  |  | ● |  |  |  |  |  |  |  |  |  |  |  |  |  |
| Peripheral blood |  |  |  |  |  |  |  |  |  |  | ● |  |  |  |  |  |  |  |  |  |  |  |  | ● |
| Daily symptoms |  |  |  |  |  |  |  |  |  |  | ● | ● | ● | ● | ● | ● | ● | ● | ● | ● | ● | ● | ● | ● |
| Antigen |  |  |  |  |  |  |  |  |  |  | ● |  | ● |  | ● |  | ● |  | ● |  | ● |  | ● | ● |

✓: house visit by mobile unit team; △: index case; ●: Household contact

**Table S2: Phylogenetic analysis**

| **Procedures**  To retrieve sequences from GISAID, the following filtering criteria were applied:   - **Location**: Asia / Thailand / Chiang Mai, Mae Hong Son, Lamphun, Lampang, Chiang Rai, and Tak - **Host**: Human - **Collection dates**: Between 1 April 2021 and 31 May 2024 - **Clade**: All - **Completeness**: Complete genomes only - **Coverage**: High coverage only   Phylogenetic reconstruction was performed using the **GTR nucleotide substitution model** in **IQ-TREE**, selected via **ModelFinder** (ref c). A **relaxed molecular clock** (mean substitution rate = 1 × 10⁻³, SD = 5 × 10⁻⁴ substitutions/site/year) was implemented in **TreeTime**, based on estimates reported by **Nextstrain** (ref d) during the same sampling period. Eight GISAID sequences were excluded prior to TreeTime analysis because they deviated significantly from the molecular clock model (i.e., identified as outliers).  We assessed multiple pairwise SNP distance thresholds previously reported in the literature (1–3). Lower thresholds appeared overly conservative, potentially missing true transmission events, while higher thresholds may have yielded likely false-positive links, given the constrained 21-day study window. |
| --- |
| **References**  1. Leducq V, Jary A, Bridier-Nahmias A, Daniel L, Zafilaza K, Damond F, et al. Nosocomial transmission clusters and lineage diversity characterized by SARS-CoV-2 genomes from two large hospitals in Paris, France, in 2020. Sci Rep. 2022 Jan 20;12(1):1094.  2. Hare D, Meaney C, Powell J, Slevin B, Brien BO, Power L, et al. Repeated transmission of SARS-CoV-2 in an overcrowded Irish emergency department elucidated by whole-genome sequencing. J Hosp Infect. 2022 Aug 1;126:1–9.  3. Campeau L, Thistlethwaite F, Yao JA, Hobbs AJ, Shahriari A, Vijh R, et al. Transmission dynamics of SARS-CoV-2 in British Columbia’s largest school district during the second half of the 2020-2021 school year. Can J Public Health Rev Can Sante Publique. 2022 Oct;113(5):653–64. |

**Table S3: Characteristics of households where all members consented to participate in the study (n = 35) and households where only some members consented (n = 58).**

| **Household characteristics** | **Households with full participation** | **Households with partial participation** |
| --- | --- | --- |
| Number of households | 35 | 58 |
| Household Size (Included index cases), median (IQR) | 4(3-5) | 5(4-6) |
| Range(min-max) | 2-7 | 3-8 |
| ≤4 persons, n(%) | 26(74) | 26 (45) |
| >4 persons, n(%) | 9(26) | 32(55) |
| Number of bedrooms, median (IQR) | 3 (2-3) | 3 (2-3) |
| 1 room,n(%) | 2(6) | 2(3) |
| 2 rooms,n(%) | 15(43) | 20(34) |
| 3 rooms,n(%) | 12(34) | 26(45) |
| 4 rooms,n(%) | 6 (17) | 8(14) |
| 5 rooms,n(%) | 0 (0) | 1(2) |
| 7 rooms,n(%) | 0 (0) | 1(2) |
| Number of bathrooms, median (IQR) | 2 (1-3) | 2 (1-2) |
| 1 room, n(%) | 12(34) | 23(40) |
| 2 rooms, n(%) | 14(40) | 24(41) |
| 3 rooms, n(%) | 6(17) | 9(16) |
| 4 rooms, n(%) | 2(6) | 2(3) |
| 5 rooms, n(%) | 1(3) | 0(0) |
| Household members wear masks indoors, n(%) | 29(9) | 48(83) |
| Air condition use | 8 (23) | 12 (21) |

**Table S4: Characteristics and laboratory results of index cases in households where all members consented to participate in the study (n = 35) and households where only some members consented (n = 58).**

| **Index case characteristics** | **Households with full participation** | | **Households with partial participation** |
| --- | --- | --- | --- |
| Number of index cases | 35 | | 58 |
| Male | 22 (63) | | 24(41) |
| Age in years, median (IQR) | 17 (11-42) | | 22 (9-43) |
| Children (<18 years) | 14 (40) | | 26 (45) |
| Adult (18:59 years) | 20 (57) | | 28 (48) |
| Elderly (>60 years) | 1 (3) | | 4 (7) |
| Highest Education level in adults, n (%) |  | |  |
| Primary school or below | 4(27) | | 6(20) |
| High school | 4 (27) | | 16(53) |
| Bachelor's degree or higher | 7(47) | | 8(27) |
| BMI^a^, n(%) |  | |  |
| Normal | 14(40) | | 27(47) |
| Underweight | 20(57) | | 29(50) |
| Overweight | 1(3) | | 2(3) |
| Have any symptoms, n (%) | 32 (91) | | 57(98) |
| Total number of SARS-CoV-2 vaccine doses received, n (%) | n=35 | | n=58 |
| None | 4 (11) | | 11(19) |
| 1-2 doses | 13 (37) | | 22(38) |
| > 3 doses | 18 (51) | | 25(43) |
| SARS-CoV-2 vaccine type received, n (%) | n=31 | | n=47 |
| mRNA only | 18 (58) | | 19(33) |
| mRNA + other | 4 (13) | | 8(14) |
| Non mRNA | 9 (29) | | 20(34) |
| Most recent vaccine type received, n (%) | n=31 | | n=47 |
| Attenuated | 1 (3) | | 0(0) |
| Vector | 1 (3) | | 8(17) |
| mRNA | 29 (94) | | 39(83) |
| Median time since last vaccine dose, n (%) | n=31 | | n=47 |
| Less than 1 month | 0 (0) | | 1(2) |
| 1-3 months | 2 (6) | | 0(0) |
| 4-6 months | 8 (26) | | 9(19) |
| More than 6 months | 21 (68) | | 37(79) |
| **Laboratory results** |  |  |  |
| Lineage of SARS CoV-2, n (%) |  | |  |
| Omicron (BA) | 23 (66) | | 33(57) |
| Omicron (XBB) | 6 (17) | | 17(29) |
| Unspecified | 6 (17) | | 8(14) |
| RT-PCR cycle thresholds |  | |  |
| N gene, median (IQR) | 25.4 (22.8 - 28.9) | | 24.8(22.2-27.9) |
| N gene < 30, n (%) | 26 (76) | | 51(87) |
| S gene, median (IQR) | 23.1(20.9-27.1) | | 22.8 (21.0 - 25.9) |
| S gene < 30, n (%) | 31 (91) | | 53(91) |

^a^Adult’s BMI: Normal (BMI: 18.5 - 22.9), underweight (BMI < 18.5) and overweight (BMI: ≥23.0); Children’s BMI: Normal (z-score: < -2), underweight (z-score: -1.5 – 1.5), and overweight (z-score: > 1.5)

**Table S5: Characteristics and laboratory results of household contacts in households where all members consented to participate in the study (n = 35) and households where only some members consented (n = 58).**

| **Household contact characteristics** | **Households with full participation** | **Households with partial participation** |
| --- | --- | --- |
| No. household members | 97 | 100 |
| Male, n (%) | 50 (52) | 55(55) |
| Age in years, median (IQR) | 38 (19 - 56) | 30(14-45) |
| Children (<18 years) | 55(57) | 50(50) |
| Adult (18:59 years) | 24(25) | 35(35) |
| Elderly (>60 years) | 18(19) | 15(15) |
| Relation with index case, n (%) |  |  |
| Child/ Grandchild | 6 (6) | 6(6) |
| Parent | 33 (34) | 31(31) |
| Grandparent | 12 (12) | 9(9) |
| Spouse | 12 (12) | 10(10) |
| Other | 34 (35) | 44(44) |
| Highest education level of adults, n (%) | n=73 | n=66 |
| Primary school or below | 21 (29) | 22(33) |
| High school | 20 (27) | 24(36) |
| Bachelor's degree or higher | 32 (44) | 20(30) |
| ^a^BMI, n (%) |  |  |
| Normal | 49 (50) | 46(46) |
| Underweight | 4 (4.1) | 6(6) |
| Overweight | 44 (45) | 48(48) |
| Have Chronic diseases, n (%) |  |  |
| Hypertension | 17 (18) | 15(15) |
| Kidney disease | 1 (1) | 1(1) |
| Diabetes | 5 (5) | 1(1) |
| Smoking, n (%) | n=73 | n=66 |
| Never | 61 (84) | 46(70) |
| Ever smoke | 4 (5) | 9(14) |
| Current smoke | 8 (11) | 11(17) |
| Total number of SARS-CoV-2 vaccine doses received, n (%) |  |  |
| 0 | 11 (11) | 11(11) |
| 1-2 doses | 23 (24) | 35(35) |
| ≥ 3 doses | 63 (65) | 54(54) |
| Vaccine type received, n (%) | n=86 | n=89 |
| mRNA only | 18 (21) | 29(33) |
| mRNA + other | 23 (27) | 16(18) |
| Non mRNA | 45 (52) | 44(49) |
| Latest vaccine type received, n (%) | n=86 | n=89 |
| Attenuated | 3 (4) | 5 (6) |
| Vector | 11 (13) | 14 (16) |
| mRNA | 72 (84) | 70 (79) |
| Time from the latest vaccine dose, n (%) | n=86 | n=89 |
| Less than 1 month | 3 (4) | 1(1) |
| 1-3 months | 6 (7) | 5(6) |
| 4-6 months | 18 (21) | 10(11) |
| More than 6 months | 59 (69) | 73(82) |
| Previously SARS CoV-2 positive, n (%) |  |  |
| Yes | 42 (43) | 53(53) |
| No | 55 (57) | 47(47) |
| Household members wear masks indoors, n (%) | 80 (82) | 84 (84) |
| **Laboratory Results** |  |  |
| NCP IgG S/CO ratio at baseline (n = 176), median (IQR) | 0.28 (0.06-0.96) | 0.43 (0.15-1.36) |
| Positive (≥1·1), n(%) | 18 (21) | 25 (27) |
| Negative (<1·1), n(%) | 67 (79) | 66 (73) |
| NCP IgM S/CO ratio at baseline (n = 176), median (IQR) | 0.12 (0.07-0.23) | 0.19 (0.08-0.35) |
| Positive (≥1·1), n(%) | 2 (2) | 3 (3) |
| Negative (<1·1), n(%) | 83 (98) | 88 (97) |

^a^Adult’s BMI: Normal (BMI: 18.5 - 22.9), underweight (BMI < 18.5) and overweight (BMI: ≥23.0); Children’s BMI: Normal (z-score: < -2), underweight (z-score: -1.5 – 1.5), and overweight (z-score: > 1.5)

**Table S6: Factors associated with household SARS CoV-2 infection based on positive RDT/PCR, at the individual level, in households where all members consented to participate in the study (n = 35) and households where only some members consented (n = 58).**

| **Characteristics** | **Events/**  **Cases** | **Households with full participation**  **n=35** | **P-value** | **Events/**  **Cases** | **Households with partial participation**  **n=56** | **P-value** |
| --- | --- | --- | --- | --- | --- | --- |
|  |  | **RR (95%CI)** |  |  | **RR (95%CI)** |  |
| **Household** |  |  |  |  |  |  |
| Household size >4 persons (Included index cases) | 4/39 | 0·90 (0·25-3·28) | 0·87 | 10/61 | 0·78 (0·31-1·99) | 0·61 |
| Number of bedrooms |  | 1·21 (0·48-3·06) | 0·68 |  | 1·10 (0·76-1·58) | 0·62 |
| Number of toilets |  | 0·59 (0·27-1·29) | 0·19 |  | 1·66 (1·03-2·68) | 0·04 |
| Have a pet | 2/27 | 0·67 (0·14-3·18) | 0·62 | 6/34 | 0·93 (0·34-2·51) | 0·89 |
| Index case wears a mask indoors | 9/83 | 1·20 (0·19-7·54) | 0·84 | 18/92 | - | - |
| Household contact wear masks indoors | 10/77 | - | - | 15/82 | 1·01 (0·25-4·01) | 0·99 |
| Separate bed with index case infected | 10/84 | - | - | 18/77 | - | - |
| Have Air conditioning | 1/16 | 0·53 (0·07-3·89) | 0·54 | 5/17 | 1·57 (0·53-4·69) | 0·42 |
| **Index cases** |  |  |  |  |  |  |
| Age group < 18 years | 6/52 | 1·14 (0·32-4·12) | 0·84 | 7/41 | 0·84 (0·32-2·18) | 0·72 |
| Male | 7/66 | 0·90 (0·23-3·50) | 0·88 | 10/41 | 1·63 (0·65-4·08) | 0·29 |
| Total vaccination number in index < 3 doses | 8/50 | 3·27 (0·68-15·81) | 0·14 | 10/47 | 1·25 (0·48-3·24) | 0·65 |
| All vaccine type received |  |  |  |  |  |  |
| mRNA + other | 1/13 | Ref |  | 2/15 | Ref |  |
| mRNA only | 5/49 | 1·38 (0·22-8·81) | 0·74 | 6/32 | 1·43 (0·34-6·06) | 0·63 |
| Non mRNA | 2/22 | 1·26 (0·16-10·18) | 0·83 | 8/38 | 1·66 (0·41-6·74) | 0·48 |
| No vaccination | 2/10 | 2·67(0·26-27·00) | 0·41 | 2/13 | 1·04 (0·18-5·85) | 0·96 |
| Latest vaccine type received |  |  |  |  |  |  |
| mRNA | 7/78 | Ref |  |  | Ref |  |
| Attenuate/Vector | 1/6 | 1·99 (0·30-13·04) | 0·47 | 1/11 | 0·41 (0·04-3·84) | 0·44 |
| No vaccination | 2/10 | 2·48 (0·29-22·90) | 0·42 | 2/13 | 0·60 (0·12-2·98) | 0·53 |
| Latest vaccine received duration |  |  |  |  |  |  |
| Less than 6 months | 2/34 | Ref |  |  | Ref |  |
| 6 months or more | 8/60 | 2·96 (0·43-20·44) | 0·27 | 13/81 | 0·58 (0·19-1·77) | 0·34 |
| Lineage of SARS CoV-2 |  |  |  |  |  |  |
| Omicron (BA) | 7/62 | Ref |  | 10/57 | Ref |  |
| Omicron (XBB) | 3/17 | 1·52 (0·48-4·86) | 0·48 | 8/28 | 1·58 (0·65-3·84) | 0·32 |
| PCR cycle thresholds (N gene) |  | 0·85 (0·73-0·99) | 0·04 |  | 0·84 (0·73-0·96) | 0·01 |
| **Household members** |  |  |  |  |  | |
| Age group |  |  |  |  |  |  |
| Children (<18 years) | 2/24 | Ref |  | 8/33 | Ref |  |
| Adult (18:59 years) | 6/52 | 1·48 (0·32-6·81) | 0·61 | 5/50 | 0·52 (0·23-1·18) | 0·12 |
| Elderly (>60 years | 2/18 | 1·34 (0·25-7·25) | 0·73 | 5/15 | 1·58 (0·74-3·35) | 0·24 |
| Male | 4/49 | 0·60 (0·16-2·33) | 0·46 | 12/54 | 1·41 (0·64-3·08) | 0·39 |
| BMI^a^ |  |  |  |  |  |  |
| Normal | 4/48 | Ref |  |  | Ref |  |
| Underweight | 1/4 | 4·01 (0·74-21·61) | 0·11 |  | 0·80 (0·12-5·49) | 0·82 |
| Overweight | 5/42 | 1·94 (0·68-5·54) | 0·22 |  | 1·03 (0·52-2·06) | 0·93 |
| Education level in adults |  |  |  |  |  |  |
| Under High school | 5/39 | 1·32 (0·50-3·46) | 0·58 | 8/46 | 1·00 (0·53-1·85) | 0·99 |
| High school or higher |  | Ref |  |  | Ref |  |
| SARS CoV-2 IgG positivity | 0/17 | - | - | 1/25 | 0·44 (0·26-0·76) | 0·0034 |
| Total vaccination number < 3 doses | 5/60 | 1·62 (0·58-4·52) | 0·36 | 7/45 | 0·70 (0·33-1·52) | 0·37 |
| All vaccine type received |  |  |  |  |  |  |
| mRNA + other | 3/21 | Ref |  | 3/16 | Ref |  |
| mRNA only | 3/18 | 1·07 (0·25-5·06) | 0·92 | 7/28 | 1·10 (0·43-2·86) | 0·84 |
| Non mRNA | 3/44 | 0·48 (0·12-1·94) | 0·30 | 6/43 | 0·73 (0·28-1·92) | 0·52 |
| No vaccination | 1/11 | 0·58 (0·06-5·79) | 0·64 | 2/11 | 0·79 (0·19-3·33) | 0·75 |
| Latest vaccine type received |  |  |  |  |  |  |
| mRNA | 9/69 | Ref |  |  | Ref |  |
| Attenuate/Vector | 0/14 | - | - | 2/19 | 0·52 (0·15-1·78) | 0·30 |
| No vaccination | 1/11 | 0·61 (0·06-6·05) | 0·67 | 2/11 | 0·77 (0·16-3·72) | 0·74 |
| Latest vaccine received duration |  |  |  |  |  |  |
| Less than 6 months |  | Ref |  |  |  |  |
| 6 months or more | 7/67 | 0·88 (0·24-3·14) | 0·84 | 15/82 | 1·13 (0·41-3·15) | 0·81 |
| Previous SARS CoV-2 positive | 4/40 | 0·92 (0·26-3·30) | 0·90 | 7/53 | 0·60 (0·24-1·49) | 0·27 |
| Ever smoked | 4/36 | 1·05 (0·25-4·39) | 0·95 | 13/53 | 1·98 (1·00-3·91) | 0·05 |
| Relation with index case |  |  |  |  |  |  |
| Spouse | 1/11 | Ref |  | 1/10 | Ref |  |
| Child/ Grandchild | 1/6 | 1·66 (0·12-21·88) | 0·70 | 1/6 | 2·11 (0·23-19·17) | 0·51 |
| Father/ Mother | 2/31 | 0·64 (0·08-5·27) | 0·68 | 6/31 | 1·93 (0·29-12·78) | 0·50 |
| Grandfather/ Grandmother | 2/12 | 1·57 (0·20-12·08) | 0·66 | 1/9 | 1·10 (0·11-10·92) | 0·93 |
| Other | 4/34 | 1·10 (0·16-7·49) | 0·92 | 9/42 | 2·02 (0·28-14·36) | 0·48 |

^a^Adult’s BMI: Normal (BMI: 18·5 - 22·9), underweight (BMI < 18·5) and overweight (BMI: ≥23·0); Children’s BMI: Normal (z-score: < -2), underweight (z-score: -1·5 – 1·5), and overweight (z-score: > 1·5)

**Table S7: Secondary attack rate in households where all members consented to participate in the study (n = 35) and households where only some members consented (n = 58).**

| **Type of transmission** | **Households with full participation** | | **Households with partial participation** | |
| --- | --- | --- | --- | --- |
|  | **n/N** | **% (95% CI)** | **n/N** | **% (95% CI)** |
| Secondary attack rate: Main analysis based on RT-PCR result |  |  |  |  |
| Household level | 10/35 | 29(15-46) | 21/58 | 36 (24-50) |
| Individual level | 18/97 | 19 (11-34) | 26/100 | 27 (19-39) |
| Secondary attack rate: Sensitivity analysis based on RT-PCR or IgM |  |  |  |  |
| Household level | 11/35 | 31 (17-49) | 22/58 | 38 (26-52) |
| Individual level | 19/97 | 20 (11-34) | 27/100 | 28(20-40) |
| Secondary attack rate: Sensitivity analysis based on phylogenetic analysis |  |  |  |  |
| Household level | 8/35 | 23(10-40) | 15/58 | 26 (15-39) |
| Individual level | 10/94 | 11 (6-21) | 18/98 | 18 (8-38) |

**Table S8: Characteristics and laboratory results of SARS-CoV-2 PCR-positive (n = 44) and SARS-CoV-2 PCR-negative household contact (n = 153).**

| **Household members characteristics** | **SARS-CoV-2 PCR**  **Positive** | **SARS-CoV-2 PCR**  **Negative** |
| --- | --- | --- |
| No. household members | 44 | 153 |
| Male | 25 (57) | 80 (52) |
| Age in years, median (IQR) |  |  |
| Children (<18 years), n (%) | 20 (46) | 85 (56) |
| Adult (18:59 years), n (%) | 12 (27) | 47 (31) |
| Elderly (>60 years), n (%) | 12 (27) | 21 (14) |
| Relation with index case |  |  |
| Child/ Grandchild | 2 (4) | 10 (6) |
| Parent | 16 (36) | 48 (31) |
| Grandparent | 7 (16) | 14 (9) |
| Spouse | 3 (7) | 19 (12) |
| Other | 16 (36) | 62 (40) |
| Highest education level of adults, n (%) |  |  |
| Primary school or below | 13 (41) | 30 (28) |
| High school | 10 (31) | 34 (32) |
| Bachelor's degree or higher | 9 (28) | 43 (40) |
| ^a^BMI, n (%) |  |  |
| Normal | 18 (41) | 77 (50) |
| Underweight | 3 (7) | 7 (5) |
| Overweight | 23 (52) | 69 (45) |
| Have Chronic diseases, n (%) |  |  |
| Hypertension | 12 (27) | 20 (13) |
| Kidney disease | 0 (0) | 2 (1) |
| Diabetes | 0 (0) | 6 (4) |
| Smoking, n (%) |  |  |
| Never | 3 (9) | 16 (15) |
| Ever smoke | 5 (16) | 8 (7) |
| Current smoke | 3 (9) | 16 (15) |
| Total number of SARS-CoV-2 vaccine doses received, n (%) |  |  |
| 0 | 3 (7) | 19 (12) |
| 1-2 doses | 14 (32) | 44 (29) |
| ≥ 3 doses | 27 (61) | 90 (59) |
| Vaccine type received, n (%) |  |  |
| mRNA only | 12 (29) | 35 (26) |
| mRNA + other | 12 (29) | 27 (20) |
| Non mRNA | 17 (41) | 72 (54) |
| Latest vaccine type received, n (%) |  |  |
| Attenuated | 1 (2) | 7 (5) |
| Vector | 6 (15) | 19 (14) |
| mRNA | 34 (83) | 108 (81) |
| Time from the latest vaccine dose, n (%) |  |  |
| Less than 1 month | 1 (2) | 3 (2) |
| 1-3 months | 2 (5) | 9 (7) |
| 4-6 months | 7 (17) | 21 (16) |
| More than 6 months | 31 (76) | 101 (75) |
| Previously SARS CoV-2 positive, n (%) |  |  |
| Yes | 41 (93) | 123 (80) |
| No | 3 (7) | 30 (20) |
| Household members wear masks indoors, n(%) | 41 (93) | 123 (80) |
| **Laboratory Results** |  |  |
| NCP IgG S/CO ratio at baseline (n = 176), median (IQR) | 0.11 (0.06-0.36) | 0.52 (0.14-1.32) |
| Positive (≥1·1), n (%) | 2 (5) | 41 (31) |
| Negative (<1·1), n (%) | 40 (95) | 93 (69) |
| NCP IgM S/CO ratio at baseline (n = 176, median (IQR) | 0.09 (0.04-0.25) | 0.15 (0.08-0.33) |
| Positive (≥1·1), n (%) | 2 (5) | 3 (2) |
| Negative (<1·1), n (%) | 40 (95) | 131 (98) |

^a^Adult’s BMI: Normal (BMI: 18.5 - 22.9), underweight (BMI < 18.5) and overweight (BMI: ≥23.0); Children’s BMI: Normal (z-score: < -2), underweight (z-score: -1.5 – 1.5), and overweight (z-score: > 1.5)

**Table S9:** **Factors associated with household SARS CoV-2 infection based on positive RDT/PCR****, at the individual level (n=197 household members at risk, 44 events)**

| **Characteristics** | **Events/**  **Cases** | **Univariable** | **P-value** | **Multivariable** | **P-value** |
| --- | --- | --- | --- | --- | --- |
|  |  | **RR (95%CI)** |  | **aRR (95%CI)** |  |
| **Household** |  |  |  |  |  |
| Household size >4 persons (Included index cases) | 21/101 | 0·90 (0·48-1·69) | 0·75 | 1·34 (0·74-2·43) | 0·33 |
| Number of bedrooms |  | 1·12 (0·88-1·42) | 0·35 |  |  |
| Number of toilets |  | 1·10 (0·80-1·52) | 0·56 |  |  |
| Have a pet | 14/64 | 1·11 (0·54-2·07) | 0·78 |  |  |
| Index case wearing masks indoors | 42/180 | 2·40 (0·39-14·78) | 0·34 |  |  |
| Household contacts wearing masks indoors | 41/164 | 2·37 (0·59-9·53) | 0·22 |  |  |
| Separate bed with infected index case | 42/166 | 3·90 (0·57-26·81) | 0·17 |  |  |
| Air condition use | 9/34 | 1·16 (0·53-2·52) | 0·72 |  |  |
| **Index cases** |  |  |  |  |  |
| Age group < 18 years | 25/96 | 1·26 (0·67-2·40) | 0·47 | 1·38 (0·79-2·41) | 0·26 |
| Male | 25/108 | 1·07 (0·57-1·99) | 0·84 |  |  |
| Total vaccine doses in index < 3 doses | 28/98 | 1·63 (0·83-3·19) | 0·15 |  |  |
| All vaccine type received |  |  |  |  |  |
| mRNA + other | 4/29 | Ref |  |  |  |
| mRNA only | 23/84 | 1·84 (0·69-4·90) | 0·22 |  |  |
| Non mRNA | 13/61 | 1·44 (0·50-4·17) | 0·50 |  |  |
| No vaccination | 4/23 | 1·03 (0·27-3·90) | 0·96 |  |  |
| Latest vaccine type received |  |  |  |  |  |
| mRNA | 37/157 | Ref |  |  |  |
| Inactivated | 2/3 | 5·85 (3·70-9·25) | <0·0001 |  |  |
| Vector | 1/14 | 0·28 (0·03-2·45) | 0·25 |  |  |
| No vaccination | 4/23 | 0·65 (0·20-2·03) | 0·51 |  |  |
| Latest vaccine received duration |  |  |  |  |  |
| Less than 6 months | 11/51 | Ref |  |  |  |
| 6 months or more | 33/146 | 1·00 (0·47-2·10) | 0·99 |  |  |
| Lineage of SARS CoV-2 |  |  |  |  |  |
| Omicron (BA) | 29/122 | Ref |  |  |  |
| Omicron (XBB) | 14/46 | 1·32 (0·71-2·46) | 0·38 |  |  |
| PCR cycle thresholds (N gene) |  | 0·92 (0·85-0·99) | 0·042 | 0·92 (0·85-1·00) | 0·045 |
| **Household contacts** |  |  |  |  | |
| Age group |  |  |  |  |  |
| Children (<18 years) | 12/58 | Ref |  | Ref |  |
| Adult (18:59 years) | 20/106 | 1·00 (0·60-1·66) | 0·99 | 1·05(0·61-1·80) | 0·86 |
| Elderly (>60 years) | 12/33 | 1·81 (1·02-3·22) | 0·044 | 1·71 (0·97-2·99) | 0·062 |
| Male | 25/105 | 1·10 (0·71-1·70) | 0·99 |  |  |
| BMI^a^ |  |  |  |  |  |
| Normal | 18/95 | Ref |  |  |  |
| Underweight | 3/10 | 1·80 (0·69-4·65) | 0·23 |  |  |
| Overweight | 23/92 | 1·60 (1·01-2·55) | 0·046 |  |  |
| Education level in adults |  |  |  |  |  |
| Under High school | 23/87 | 1·36 (0·90-2·06) | 0·14 |  |  |
| High school or higher | 21/110 | Ref |  |  |  |
| SARS CoV-2 IgG positivity | 2/43 | 0·44 (0·28-0·70) | 0·00045 | 0·45 (0·28-0·71) | 0·001 |
| Total vaccine doses in index < 3 doses | 17/80 | 0·89 (0·55-1·42) | 0·62 |  |  |
| All vaccine type received |  |  |  |  |  |
| mRNA + other | 12/39 | Ref |  |  |  |
| mRNA only | 12/47 | 0·84 (0·40-1·57) | 0·49 |  |  |
| Non mRNA | 17/89 | 0·66 (0·37-1·19) | 0·17 |  |  |
| No vaccination | 3/22 | 0·42 (0·20-1·20) | 0·12 |  |  |
| Latest vaccine type received |  |  |  |  |  |
| mRNA | 34/142 | Ref |  |  |  |
| Inactivated | 1/8 | 0·40 (0·10-1·53) | 0·18 |  |  |
| Vector | 6/25 | 1·00 (0·42-2·41) | 0·99 |  |  |
| No vaccination | 3/22 | 0·45 (0·14-1·47) | 0·19 |  |  |
| Latest vaccine received duration |  |  |  |  |  |
| Less than 6 months | 10/45 | Ref |  |  |  |
| 6 months or more | 34/154 | 1·02 (0·58-1·78) | 0·95 |  |  |
| Previous SARS CoV-2 positive | 16/95 | 0·63 (0·34-1·20) | 0·13 |  |  |
| Ever smoked | 20/90 | 1·03 (0·67-1·59) | 0·89 |  |  |
| Relation with index case |  |  |  |  |  |
| Spouse | 3/22 | Ref |  |  |  |
| Child/ Grandchild | 2/12 | 1·36 (0·38-4·89) | 0·64 |  |  |
| Father/ Mother | 16/64 | 1·62 (0·65-4·07) | 0·30 |  |  |
| Grandfather/ Grandmother | 7/21 | 2·00 (0·68-5·88) | 0·20 |  |  |
| Other | 16/78 | 1·21 (0·47-3·08) | 0·70 |  |  |

^a^Adult’s BMI: Normal (BMI: 18·5 - 22·9), underweight (BMI < 18·5) and overweight (BMI: ≥23·0); Children’s BMI: Normal (z-score: < -2), underweight (z-score: -1·5 – 1·5), and overweight (z-score: > 1·5)

**Table S10: Factors associated with household SARS-CoV-2 infection based on positive RDT/PCR or positive SARS CoV-2 IgM, at individual level (n=197 household members at risk and 46 events)**

| **Characteristics** | **Events/**  **Cases** | **Univariable** | **P-value** | **Multivariable** | | **P-value** |
| --- | --- | --- | --- | --- | --- | --- |
|  |  | **RR (95%CI)** |  | **aRR (95%CI)** | |  |
| **Household** |  |  |  | |  |  |
| Household size >4 persons (Including index cases) | 23/101 | 0·90 (0·48-1·69) | 0·75 | | 1·36 (0·76-2·47) | 0·30 |
| Number of bedrooms |  | 1·13 (0·90-1·41) | 0·30 | |  |  |
| Number of toilets |  | 1·17 (0·85-1·61) | 0·33 | |  |  |
| Have a pet | 14/64 | 0·98 (0·50-1·91) | 0·96 | |  |  |
| Index case wearing masks indoors | 44/180 | 2·47 (0·40-15·11) | 0·33 | |  |  |
| Household contacts wearing masks indoors | 41/164 | 1·52 (0·55-4·19) | 0·42 | |  |  |
| Separate bed with infected index case | 44/166 | 4·08 (0·59-27·98) | 0·15 | |  |  |
| Air condition use | 9/34 | 1·10 (0·51-2·39) | 0·81 | |  |  |
| **Index cases** | 26/96 | 1·30 (0·71-2·37) | 0·40 | |  |  |
| Age group < 18 years | 25/108 | 0·98 (0·54-1·78) | 0·94 | | 1·44 (0·83-2·50) | 0·20 |
| Male | 23/101 | 0·90 (0·48-1·69) | 0·75 | |  |  |
| Total vaccine doses in index < 3 doses | 29/98 | 1·66 (0·87-3·17) | 0·12 | |  |  |
| All vaccine type received |  |  |  | |  |  |
| mRNA + other | 4/29 | Ref |  | |  |  |
| mRNA only | 24/84 | 1·91 (0·72-5·04) | 0·19 | |  |  |
| Non mRNA | 13/61 | 1·46 (0·50-4·20) | 0·49 | |  |  |
| No vaccination | 5/23 | 1·59 (0·41-4·71) | 0·45 | |  |  |
| Latest vaccine type received |  |  |  | |  |  |
| mRNA | 38/157 | Ref |  | |  |  |
| Inactivated | 2/3 | 5·78 (3·68-9·06) | <0·0001 | |  |  |
| Vector | 1/14 | 0·28 (0·03-2·37) | 0·24 | |  |  |
| No vaccination | 5/23 | 0·80 (0·25-2·55) | 0·70 | |  |  |
| Latest vaccine received duration |  |  |  | |  |  |
| Less than 6 months | 12/51 | Ref |  | |  |  |
| 6 months or more | 34/146 | 0·98 (0·49-1·97) | 0·96 | |  |  |
| Lineage of SARS CoV-2 |  |  |  | |  |  |
| Omicron (BA) | 31/122 | Ref |  | |  |  |
| Omicron (XBB) | 14/46 | 1·23 (0·67-2·26) | 0·51 | |  |  |
| PCR cycle thresholds (N gene) |  | 0·93 (0·86-1·00) | 0·060 | | 0·93 (0·86-1·00) | 0·052 |
| **Household contacts** |  |  |  | |  | |
| Age, year |  | 1·01 (0·98-1·02) | 0·13 | |  |  |
| Age group |  |  |  | |  |  |
| Children (<18 years) | 12/58 | Ref |  | | Ref |  |
| Adult (18:59 years) | 22/106 | 1·064 (0·64-1·75) | 0·81 | | 1·01 (0·59-1·75) | 0·96 |
| Elderly (>60 years) | 12/33 | 1·80 (1·01-3·22) | 0·046 | | 1·70 (0·97-2·98) | 0·066 |
| Male | 26/105 | 1·08 (0·70-1·67) | 0·71 | |  |  |
| BMI^a^ |  |  |  | |  |  |
| Normal | 18/95 | Ref |  | |  |  |
| Underweight | 3/10 | 1·50 (0·55-4·13) | 0·42 | |  |  |
| Overweight | 23/92 | 1·39 (0·88-2·21) | 0·16 | |  |  |
| Education level in adults |  |  |  | |  |  |
| Under High school | 22/110 | Ref |  | |  |  |
| High school or higher | 24/87 | 1·36 (0·90-2·06) | 0·14 | |  |  |
| SARS CoV-2 IgG positivity | 4/43 | 0·61 (0·41-0·91) | 0·015 | | 0·61 (0·40-0·92) | 0·019 |
| Total vaccine doses in index < 3 doses | 18/80 | 0·91 (0·58-1·44) | 0·69 | |  |  |
| All vaccine type received |  |  |  | |  |  |
| mRNA + other | 13/39 | Ref |  | |  |  |
| mRNA only | 12/47 | 0·76 (0·41-1·41) | 0·39 | |  |  |
| Non mRNA | 18/89 | 0·63 (0·35-1·11) | 0·11 | |  |  |
| No vaccination | 3/22 | 0·38 (0·13-1·14) | 0·084 | |  |  |
| Latest vaccine type received |  |  |  | |  |  |
| mRNA | 35/142 | Ref |  | |  |  |
| Inactivated | 2/8 | 0·40 (0·11-1·53) | 0·18 | |  |  |
| Vector | 6/25 | 1·00 (0·42-2·41) | 0·99 | |  |  |
| No vaccination | 3/22 | 0·45 (0·14-1·47) | 0·19 | |  |  |
| Latest vaccine received duration |  |  |  | |  |  |
| Less than 6 months | 10/45 | Ref |  | |  |  |
| 6 months or more | 36/154 | 1·10 (0·61-1·96) | 0·76 | |  |  |
| Previous SARS CoV-2 positive | 18/95 | 0·74 (0·40-1·36) | 0·33 | |  |  |
| Ever smoked | 21/90 | 1·04 (0·68-1·58) | 0·87 | |  |  |
| Relation with index case |  |  |  | |  |  |
| Spouse | 3/22 | Ref |  | |  |  |
| Child/ Grandchild | 2/12 | 1·40 (0·37-5·25) | 0·62 | |  |  |
| Father/ Mother | 17/64 | 1·79(0·69-4·65) | 0·23 | |  |  |
| Grandfather/ Grandmother | 7/21 | 2·12(0·70-6·42) | 0·18 | |  |  |
| Other | 17/78 | 1·33(0·50-3·50) | 0·56 | |  |  |

^a^Adult’s BMI: Normal (BMI: 18·5 - 22·9), underweight (BMI < 18·5) and overweight (BMI: ≥23·0); Children’s BMI: Normal (z-score: < -2), underweight (z-score: -1·5 – 1·5), and overweight (z-score: > 1·5)

**Table S11: Factors associated with household SARS-CoV-2 infection based on positive RDT/PCR and phylogenetic analysis, at individual level (n=192 household members at risk, 28 events)**

| **Characteristics** | **Events/**  **Cases** | **Univariable** | **P-value** | **Multivariable** | **P-value** |
| --- | --- | --- | --- | --- | --- |
|  |  | **RR (95%CI)** |  | **aRR (95%CI)** |  |
| **Household** |  |  |  |  |  |
| Household size >4 persons (Included index cases) | 14/100 | 0·93 (0·43-2·00) | 0·85 | 1·54  (0·76-3·13) | 0·22 |
| Number of bedrooms |  | 1·15 (0·8-1·62) | 0·43 |  |  |
| Number of toilets |  | 1·10 (0·81-1·62) | 0·67 |  |  |
| Have a pet | 8/61 | 0·89 (0·38-2·08) | 0·79 |  |  |
| Index case wears a mask indoors | 27/175 | 2·98 (0·48-18·33) | 0·24 |  |  |
| Household members wear masks indoors | 25/159 | 1·61 (0·38-6·76) | 0·52 |  |  |
| Air condition use | 6/33 | 1·20 (0·44-3·28) | 0·72 |  |  |
| **Index cases** |  |  |  |  |  |
| Age group < 18 years | 13/93 | 0·87 (0·40-1·89) | 0·73 | 1·03 (0·51-2·08) | 0·93 |
| Male | 17/107 | 1·18 (0·55-2·53) | 0·66 |  |  |
| Total vaccination number in index < 3 doses | 18/97 | 1·59 (0·70-3·61) | 0·27 |  |  |
| All vaccine type received |  |  |  |  |  |
| mRNA + other | 3/28 | Ref |  |  |  |
| mRNA only | 11/81 | 1·32 (0·41-4·23) | 0·64 |  |  |
| Non mRNA | 10/60 | 1·65 (0·50-5·43) | 0·41 |  |  |
| No vaccination | 4/23 | 1·52 (0·37-6·20) | 0·56 |  |  |
| Latest vaccine type received |  |  |  |  |  |
| mRNA | 22/152 | Ref |  |  |  |
| Inactivated/Vector | 2/17 | 0·74 (0·16-3·42) | 0·70 |  |  |
| No vaccination | 4/23 | 1·07 (0·29-3·97) | 0·92 |  |  |
| Latest vaccine received duration, day |  |  |  |  |  |
| Less than 6 months | 7/51 | Ref |  |  |  |
| 6 months or more | 21/141 | 1·09 (0·39-3·01) | 0·87 |  |  |
| Lineage of SARS CoV-2 |  |  |  |  |  |
| Omicron (BA) | 17/119 | Ref |  |  |  |
| Omicron (XBB) | 11/45 | 1·65 (0·80-3·42) | 0·18 |  |  |
| PCR cycle thresholds (N gene) |  | 0·84 (0·76-0·93) | <0·0001 | 0·82 (0·74-0·92) | <0·0001 |
| **Household contacts** |  |  |  |  | |
| Age, year |  | 0·86 (0·47-1·58) | 0·64 |  |  |
| Age group |  |  |  |  |  |
| Children (<18 years) | 10/57 | Ref |  | Ref |  |
| Adult (18:59 years) | 11/102 | 0·71 (0·38-1·42) | 0·34 | 0·93 (0·45-1·92) | 0·84 |
| Elderly (>60 years) | 7/33 | 1·32 (0·65-2·66) | 0·45 | 1·75 (0·90-3·40) |  |
| Male | 16/103 | 1·06 (0·55-2·06) | 0·86 |  |  |
| BMI^a^ |  |  |  |  |  |
| Normal | 12/93 | Ref |  |  |  |
| Underweight | 2/10 | 1·66 (0·46-5·96) | 0·44 |  |  |
| Overweight | 14/89 | 1·35 (0·73-2·48) | 0·34 |  |  |
| Education level in adults |  |  |  |  |  |
| Under High school | 15/107 | Ref |  |  |  |
| High school or higher | 13/85 | 1·09 (0·63-1·89) | 0·75 |  |  |
| SARS CoV-2 IgG positivity | 1/42 | 0·39 (0·21-0·74) | 0·004 | 0·42  (0·22-0·83) | 0·013 |
| Total vaccine dose < 3 doses | 12/79 | 1·00 (0·54-1·86) | 0·99 |  |  |
| All vaccine type received |  |  |  |  |  |
| mRNA + other | 6/37 | Ref |  |  |  |
| mRNA only | 10/46 | 1·17 (0·51-2·68) | 0·70 |  |  |
| Non mRNA | 9/87 | 0·64 (0·27-1·49) | 0·30 |  |  |
| No vaccination | 3/22 | 0·72 (0·21-2·48) | 0·60 |  |  |
| Latest vaccine type received |  |  |  |  |  |
| mRNA | 23/137 | Ref |  |  |  |
| Inactivated/Vector | 2/33 | 0·34 (0·09-1·32) | 0·12 |  |  |
| No vaccination | 3/22 | 0·70 (0·20-2·46) | 0·58 |  |  |
| Latest vaccine received duration, day |  |  |  |  |  |
| Less than 6 months | 6/43 | Ref |  |  |  |
| 6 months or more | 22/151 | 0·98 (0·46-2·12) | 0·97 |  |  |
| Previous SARS CoV-2 positive | 11/80 | 0·72 (0·35-1·48) | 0·37 |  |  |
| Ever smoked | 17/89 | 1·65 (0·86-3·15) | 0·13 |  |  |
| Relation with index case |  |  |  |  |  |
| Spouse | 2/21 | Ref |  |  |  |
| Child/ Grandchild | 2/12 | 1·66 (0·33-8·32) | 0·54 |  |  |
| Father/ Mother | 8/62 | 1·19 (0·35-4·07) | 0·78 |  |  |
| Grandfather/ Grandmother | 3/21 | 1·28 (0·31-5·25) | 0·73 |  |  |
| Other | 13/76 | 1·45 (0·43-4·97) | 0·55 |  |  |

^a^Adult’s BMI: Normal (BMI: 18·5 - 22·9), underweight (BMI < 18·5) and overweight (BMI: ≥23·0); Children’s BMI: Normal (z-score: < -2), underweight (z-score: -1·5 – 1·5), and overweight (z-score: > 1·5)

**Figure S1: Phylogenetic tree of SARS-CoV-2 sequences from the study and GISAID**


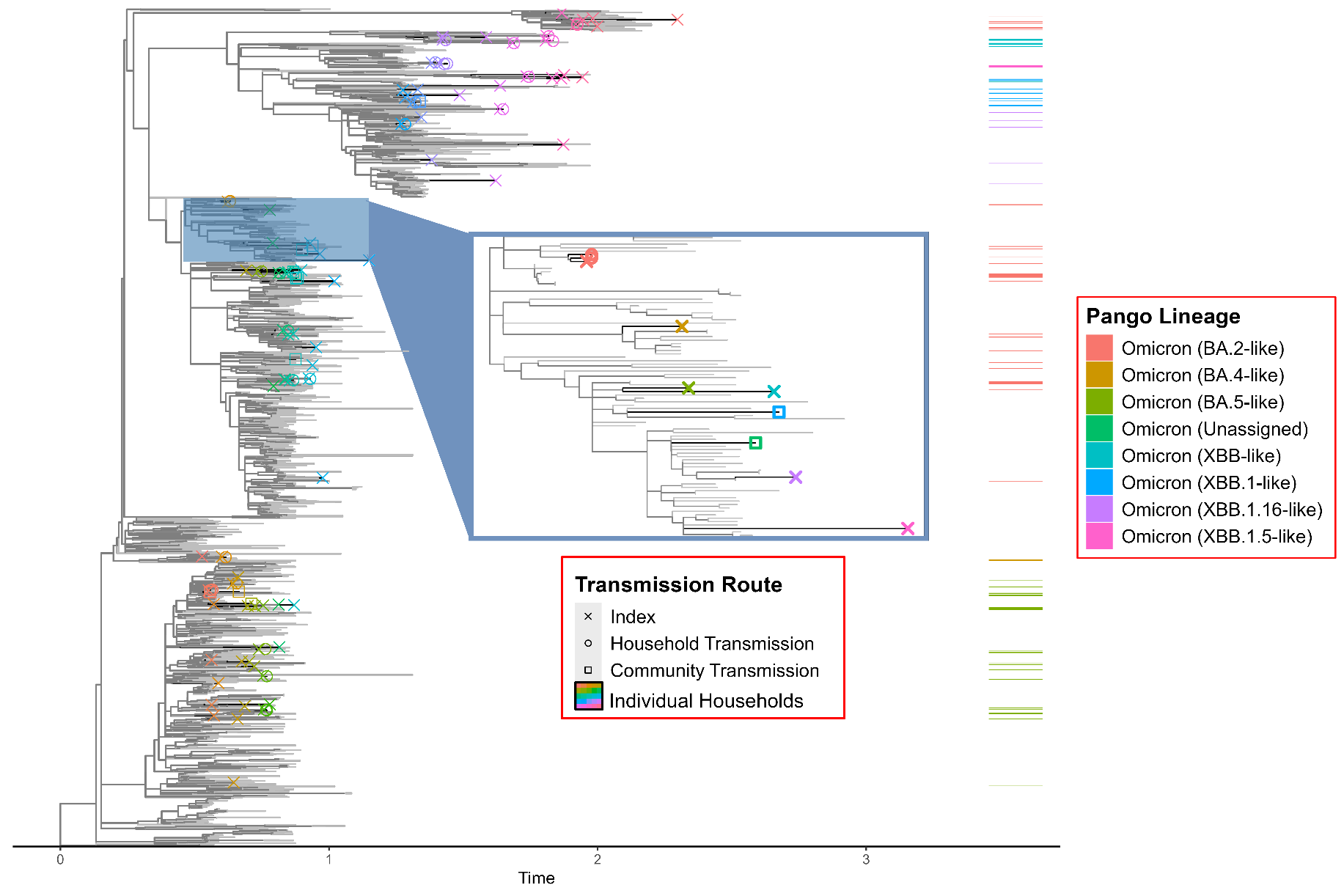
Maximum-likelihood phylogenetic tree of 121 SARS-CoV-2 genomes from this study (81 index cases and 40 contacts) and 1,850 contemporaneous sequences from GISAID collected in northern Thailand. GISAID sequences are shown in grey. A highlighted subset of the tree (blue) illustrates inferred transmission routes. Colors of the tip points indicate different households; squares denote intra-household transmission, and circles denote community-acquired infection. Pango lineages of study sequences are shown on the right, with a corresponding set of colors.
