# Supplementary material for: Household SARS-CoV-2 transmission during Omicron wave in Chiang Mai, Thailand: a prospective observational study
Source: Lancet Reg Health Southeast Asia. 2026 Jan 5;44:100711. doi: 10.1016/j.lansea.2025.100711 (PMC12810560; doi:10.1016/j.lansea.2025.100711)
Supplement: Abstract [file mmc2.docx]

***Disclaimer***

***This translation in Thai was submitted by the authors and we reproduce it as supplied. It has not been peer reviewed. Our editorial processes have only been applied to the original abstract in English, which should serve as reference for this manuscript.*”**

**บทคัดย่อ**

**บทนำ:** การศึกษาการถ่ายทอดเชื้อไวรัส SARS-CoV-2 ในเด็กในประเทศไทยมีค่อนข้างจำกัดและมีในเฉพาะช่วงการระบาดระลอกแรกๆ ของสายพันธุ์อัลฟาและเดลต้า การศึกษานี้จึงมีวัตถุเพื่อศึกษาการถ่ายทอดเชื้อภายในครัวเรือน ในจังหวัดเชียงใหม่ ทางภาคเหนือของประเทศไทย ในช่วงการระบาดของสายพันธุ์โอมิครอน และหลังการเริ่มนโยบายการให้วัคซีนของประเทศ

**วิธีการ:** การศึกษาเชิงสังเกตการณ์แบบไปข้างหน้า (Prospective observational study) นี้ได้รับสมัครครัวเรือนที่มีผู้ป่วย COVID-19 ที่ได้รับการยืนยันอย่างน้อย 1 ราย เป็นผู้ป่วยเริ่มแรก (Index case) และผู้สัมผัสใกล้ชิดที่ไม่ติดเชื้อ (Contract) อย่างน้อย 1 ราย โดยครัวเรือนนั้นต้องมีเด็กอายุต่ำกว่า 18 ปี อาศัยอยู่ในบ้านด้วย ซึ่งอาจเป็นผู้ป่วยเริ่มแรกหรือผู้สัมผัสก็ได้ ข้อมูลของผู้เข้าร่วมการศึกษา ตัวอย่างสารคัดหลั่งจากโพรงจมูก และตัวอย่างเลือดถูกเก็บในนัดหมายแรกและนัดหมายสุดท้าย ผู้เข้าร่วมการศึกษาบันทึกอาการด้วยตนเองทุกวันเป็นเวลา 21 วัน และทำการทดสอบแอนติเจนของไวรัส SARS-CoV-2 ด้วยตนเองทุกๆ สองวันเป็นเวลา 2 สัปดาห์ การติดเชื้อได้รับการยืนยันโดย RT-PCR มีการคำนวณอัตราป่วยระลอกสอง (Secondary attack rates) และวิเคราะห์ปัจจัยที่เกี่ยวข้องโดยใช้แบบจำลองหลายตัวแปร (Generalized estimating equations, GEE) การถ่ายทอดเชื้อภายในครัวเรือนถูกยืนยันโดยการวิเคราะห์ Phylogenetic analysis

**ผลการศึกษา:** ระหว่างเดือนกรกฎาคม 2565 ถึงพฤษภาคม 2567 มีครัวเรือนเข้าร่วมการศึกษาทั้งสิ้น 93 ครัวเรือน ประกอบด้วยผู้ป่วยเริ่มแรก 93 ราย และผู้สัมผัสใกล้ชิด 197 ราย โดยพบว่า 52% ของผู้ป่วยเริ่มแรก และ 29% ของผู้สัมผัสใกล้ชิดมีอายุต่ำกว่า 18 ปี มีผู้สัมผัสใกล้ชิดจำนวน 44 รายติดเชื้อ SARS-CoV-2 แม้ว่าร้อยละ 90 จะเคยได้รับวัคซีนแล้ว (โดยในจำนวนนี้ 75% ได้รับวัคซีนมากกว่า 6 เดือนก่อนหน้า) ส่งผลให้อัตราการติดเชื้อระลอกสองของครัวเรือน (household secondary attack rate; SAR) อยู่ที่ 33% (95% confidence interval, CI: 24–44) อย่างไรก็ตาม เมื่อพิจารณาเฉพาะการถ่ายทอดเชื้อที่ได้รับการยืนยันด้วยการวิเคราะห์ phylogenetic analysis พบว่าอัตราการติดเชื้อระลอกสองของครัวเรือนอยู่ที่ 25% (95% CI: 17–35) ปัจจัยที่สัมพันธ์กับการถ่ายทอดเชื้อในครัวเรือนที่ลดลงอย่างมีนัยสำคัญ ได้แก่ ปริมาณไวรัสที่ต่ำในผู้ป่วยเริ่มแรก (adjusted relative risk, aRR: 0.82; 95% CI: 0.74–0.92) และการที่ผู้สัมผัสใกล้ชิดมีผลตรวจ IgG ต่อ nucleocapsid protein (anti-NCP IgG) เป็นบวกในวันแรกของการติดตาม (aRR: 0.42; 95% CI: 0.22–0.83)

**การอภิปรายผล:** แม้จะมีการให้วัคซีนอย่างแพร่หลาย การถ่ายทอดเชื้อ SARS-CoV-2 ภายในครัวเรือนยังคงพบได้ทั่วไป ภูมิคุ้มกันในผู้สัมผัสและปริมาณไวรัสที่ต่ำในผู้ป่วยเริ่มแรกช่วยลดความเสี่ยงในการติดเชื้อ ผลการวิจัยเหล่านี้เน้นย้ำถึงบทบาทสำคัญของครัวเรือนในการแพร่เชื้อของโรคระบาดอย่างต่อเนื่อง และเน้นย้ำถึงคุณค่าของการฉีดวัคซีนเพื่อกระตุ้นภูมิคุ้มกัน และการเฝ้าระวังโดยใช้ข้อมูลพันธุกรรมของไวรัสช่วยระบุเส้นทางการแพร่เชื้อ และเป็นประโยชน์ต่อการกำหนดและจัดการนโยบายการป้องกันการระบาดของ SARS-CoV-2

**คำสำคัญ:** SARS-CoV-2; การถ่ายทอดเชื้อในครัวเรือน; สายพันธุ์โอมิครอน; anti-NCP IgG; การหาลำดับจีโนมทั้งหมด; ประเทศไทย; เด็ก
